# Conflict monitoring and emotional processing in 3,4-methylenedioxymethamphetamine (MDMA) and methamphetamine users – A comparative neurophysiological study

**DOI:** 10.1016/j.nicl.2024.103579

**Published:** 2024-02-15

**Authors:** Antje Opitz, Josua Zimmermann, David M. Cole, Rebecca C. Coray, Anna Zachäi, Markus R. Baumgartner, Andrea E. Steuer, Maximilian Pilhatsch, Boris B. Quednow, Christian Beste, Ann-Kathrin Stock

**Affiliations:** a Cognitive Neurophysiology, Department of Child and Adolescent Psychiatry, Faculty of Medicine, TU Dresden, Germany; b Experimental and Clinical Pharmacopsychology, Department of Adult Psychiatry and Psychotherapy, Psychiatric University Hospital Zurich, University of Zurich, Switzerland; c Neuroscience Center Zurich, University of Zurich and ETH Zurich, Switzerland; d Translational Psychiatry Lab, University Psychiatric Clinics Basel, University of Basel, Basel, Switzerland; e Center for Forensic Hair Analytics, Institute of Forensic Medicine, University of Zurich, Zurich, Switzerland; f Department of Forensic Pharmacology & Toxicology, Zurich Institute of Forensic Medicine, University of Zurich, 8057 Zurich, Switzerland; g Department of Psychiatry and Psychotherapy, Carl Gustav Carus University Hospital, TU Dresden, Dresden, Germany; h Department of Psychiatry and Psychotherapy, Elblandklinikum, Radebeul, Germany; i Biopsychology, Department of Psychology, School of Science, TU Dresden, Germany

**Keywords:** Conflict control, ERP, MDMA, Methamphetamine, Social cognition, Stroop effect

## Abstract

In stimulant use and addiction, conflict control processes are crucial for regulating substance use and sustaining abstinence, which can be particularly challenging in social-affective situations. Users of methamphetamine (METH, “Ice”) and 3,4-methylenedioxymethamphetamine (MDMA, “Ecstasy”) both experience impulse control deficits, but display different social-affective and addictive profiles. We thus aimed to compare the effects of chronic use of the substituted amphetamines METH and MDMA on conflict control processes in different social-affective contexts (i.e., anger and happiness) and investigate their underlying neurophysiological mechanisms. For this purpose, chronic but recently abstinent users of METH (n = 38) and MDMA (n = 42), as well as amphetamine-naïve healthy controls (n = 83) performed an emotional face-word Stroop paradigm, while event-related potentials (ERPs) were recorded. Instead of substance-specific differences, both MDMA and METH users showed smaller behavioral effects of cognitive-emotional conflict processing (independently of emotional valence) and selective deficits in emotional processing of anger content. Both effects were underpinned by stronger P3 ERP modulations suggesting that users of substituted amphetamines employ altered stimulus–response mapping and decision-making. Given that these processes are modulated by noradrenaline and that both MDMA and METH use may be associated with noradrenergic dysfunctions, the noradrenaline system may underlie the observed substance-related similarities. Better understanding the functional relevance of this currently still under-researched neurotransmitter and its functional changes in chronic users of substituted amphetamines is thus an important avenue for future research.

## Highlights

• We compared the effects of chronic MDMA and methamphetamine use on conflict control.
• Users showed reduced cognitive-emotional conflict processing (compared to non-users).
• Users also showed selective deficits in emotional processing of anger content.
• Stronger P3 modulations suggest altered S-R mapping and decision-making in users.

## 1 Introduction

Increases of 3,4-methylenedioxymethamphetamine (MDMA, “Ecstasy”, “Molly”, “Emma”) purity in tablets and powder as well as indices for spreading methamphetamine (METH, “Crystal”, “Ice”, “Tina”) use in Europe put growing numbers of users at risk of harmful consequences (Office, 2022). Both MDMA and METH users experience partially similar, but also substance-specific impairments in executive functions (Fernández-Serrano et al., 2011, Potvin et al., 2018, Roberts et al., 2016). In particular, deficits in impulse control functions have been observed in both user groups (Fernández-Serrano et al., 2011, Potvin et al., 2018, Lee et al., 2019, Smith et al., 2014) and this may affect how users interact with other individuals. However, the chronic effects of MDMA and METH on impulse control in different social-affective contexts and the underlying neurophysiological mechanisms have rarely been investigated, or compared directly.

MDMA and METH primarily target monoamine reuptake mechanisms, i.e., serotonin, dopamine, and noradrenaline transporters (Hysek et al., 2011, Gouzoulis-Mayfrank and Daumann, 2009). While both substances overlap in their ability to release noradrenaline, they strongly differ in their acute effects on serotonin (mainly released by MDMA) and dopamine (mainly released by METH) (Liechti, 2015). As a consequence, chronic MDMA exposure is mainly associated with persistent changes to the serotonin system (Roberts et al., 2016), whereas chronic METH is mainly related to lasting alterations within the dopamine system (Ashok et al., 2017). Corresponding changes in noradrenaline transmission are however still under-studied. Importantly, MDMA and METH strongly differ in their addictive potential due to their different effects on the dopamine system; while MDMA addiction is very rare (Office, 2022), METH is a highly addictive substance (Paulus and Stewart, 2020).

Moreover, the monoamine systems play a crucial role in impulse control functions (Dalley and Roiser, 2012, Pattij and Vanderschuren, 2008). Specifically, dopamine and noradrenaline modulate different facets of inhibitory control (i.e., the ability to stop thoughts and actions) (Robbins and Arnsten, 2009, Yu et al., 2022, Mückschel et al., 2017). The serotonergic system seems to have little effect on motor inhibition (i.e., the inhibition of motor movements) (Bari and Robbins, 2013), but has been suggested to increase premature responding (Dalley and Roiser, 2012) and reduce cognitive inhibition (i.e., the inhibition of thoughts and conflicting information) (Quednow et al., 2012). Both chronic MDMA and METH users have shown impulse control deficits. Meta-analyses revealed impaired response inhibition/motor impulsivity in chronic (heavy) users (Lee et al., 2019, Smith et al., 2014). Similarly, cognitive inhibition/interference control was shown to be impaired in chronic METH users (Farhadian, 2017, Salo et al., 2009) and, to a lesser extent, also in chronic MDMA users (Yip and Lee, 2005, Wagner et al., 2013). Furthermore, both user groups demonstrated higher trait impulsivity in questionnaires (Hanson et al., 2008, Kogachi et al., 2017, Lee et al., 2009, Morgan, 1998). In neuroimaging studies, impulse control deficits in stimulant use disorders have been associated with hypoactivation of frontal cortical regions, including the prefrontal cortex (PFC) and anterior cingulate cortex (ACC) (Ceceli et al., 2022, Koob and Volkow, 2016) – both of which are highly innervated by monoaminergic pathways (Robbins and Arnsten, 2009).

Despite the shared characteristic of lower impulsive control, MDMA and METH users often differ in their social behavior. MDMA acutely elicits prosocial behavior (Hysek et al., 2014), but has no known chronic effects on social behavior (Carlyle et al., 2019, Wunderli et al., 2017) and only heavy use appears to be related to prolonged elevated aggression (although more recent evidence is inconsistent) (Hoshi et al., 2007, Morgan, 2000). Specifically, MDMA users show improved cognitive (Carlyle et al., 2019, Wunderli et al., 2017) and self-reported emotional empathy (Carlyle et al., 2019), while emotion processing and recognition seem to be unaffected in chronic MDMA use (Burgess et al., 2011). In contrast, METH is related to impaired social-cognitive functions (Henry et al., 2009, Homer et al., 2008, Uhlmann et al., 2018, Quednow, 2017), less prosocial behavior (Li et al., 2022), and chronically increased aggression (Lederer et al., 2016, Payer et al., 2011). Specifically, chronic METH users are compromised in their ability to respond empathically (Kim et al., 2010) and exhibit deficits in recognising emotional faces, in particular those with fear (Kim et al., 2011) and anger (Uhlmann et al., 2018) expressions.

The latter may reflect dysfunctions of the PFC due to the downregulated dopaminergic system (Harmer et al., 2001, Lawrence et al., 2002), which plays a key role in mediating aggressive behavior (Suri et al., 2015) (which has nevertheless also been linked to serotonin (da Cunha-Bang and Knudsen, 2021). Impulsive behavior is strongly linked to aggression (Brennan and Baskin-Sommers, 2019, Ramírez and Andreu, 2006). The dopamine system is involved in inhibitory control functions (Beste et al., 2016, Ullsperger, 2010) and differentially modulated by METH compared to MDMA. For these reasons, we expected inhibitory control functions to be differently affected in METH and MDMA users when anger-related content is presented/processed. METH users may respond more impulsively and may be less able to regulate conflicts in an aggressive/angry setting, whereas MDMA users may not be affected by anger-related content. Therefore, the processing of negative emotional (i.e., anger) information may require more cognitive resources in chronic METH users, which could lead to fewer available (residual) resources to solve a cognitive-emotional conflict.

In this study, we thus employed an emotional Stroop paradigm to investigate the effects of distinct social-affective content information (i.e., anger and happiness) on conflict monitoring processes, while recording event-related potentials (ERPs) in order to be able to disentangle different cognitive subprocesses. We compared cognitive-emotional conflict control (as assessed by the size of conflict effects in experimental paradigms like the Stroop task) and the neurophysiological correlates thereof between MDMA and METH users, as well as age- and sex-matched controls.

Previous findings of classical conflict tasks suggest that MDMA users might not have strong impairments in conflict control (Dafters, 2006, Piechatzek et al., 2009, Wagner et al., 2013), while METH users might have more consistent impairments in conflict control (Salo et al., 2009, Simon et al., 2000, Salo et al., 2007, Nestor et al., 2011, Proebstl et al., 2019). Conflict-related processes are reflected in the N2 ERP component, which is typically larger in conflicting than in non-conflicting situations (Folstein and Van Petten, 2007, Larson et al., 2014, Kanske and Kotz, 2011, Chmielewski and Beste, 2017, Gohil et al., 2017). Nevertheless, evidence from emotional face-word Stroop paradigms implies that neither the N2, nor other components related to early perceptual processing (like the P1 and N1 ERP) are modulated by cognitive-emotional conflict information (Pool et al., 2016, Clayson and Larson, 2013, Schreiter et al., 2018, Schreiter et al., 2018). Therefore, we do not expect the emotional Stroop effect (i.e., the performance difference between Stroop trials with conflicting affective stimuli and non-conflicting affective stimuli) to be modulated by MDMA and METH at these processing stages. Instead, we expect substance-related changes in conflict monitoring and response selection processes to emerge in later ERP components. The conflict slow potential (CSP) and P3 are expected to be of particular relevance, given previous findings on CSP and P3 modulations in Stroop paradigms (Schreiter et al., 2018, Haifeng et al., 2015, Schreiter et al., 2019), as well as the knowledge that the complexity of the administered stimuli (emotional words and facial expressions) should mainly affect later processing stages (Larson et al., 2014, Chen et al., 2016), and that the P3 has previously been shown to be altered in abstinent METH users (Haifeng et al., 2015). The CSP likely indicates an increase in the recruitment of cognitive control resources and is generally more enhanced (i.e., more positive) following conflicting situations (Larson et al., 2014, Chen et al., 2016, Zinchenko et al., 2015). The P3 likely represents stimulus–response mapping and decision making processes. It is presumably enlarged following negative compared to neutral/positive social-affective content (Schreiter et al., 2018, Kaestner and Polich, 2011) and reduced following emotionally conflicting situations (Clayson and Larson, 2013, Schreiter et al., 2018, Schreiter et al., 2019).

In summary, we hypothesized that MDMA and METH users (but not their respective controls) differ in conflict processing depending on the social-affective information: In angry conditions, METH users should behaviorally perform poorer than MDMA users, whereas we expected no such differences in happy conditions. Generally, both MDMA and METH users should show larger emotional Stroop effects (i.e., lower emotional-cognitive impulse control) than controls. These modulations should be reflected by changes in the amplitudes of the CSP and the P3 (less likely also the N2).

## 2 Materials and methods

### 2.1 Sample

N = 203 participants were recruited via online advertisements, through flyers and posters in the nightlife scene, at counselling services, addiction clinics, and public places. MDMA users and their healthy controls were mainly recruited in Zurich (Switzerland), while METH users and their healthy controls were mainly recruited in Dresden (Germany). At each site, healthy controls were matched for sex and age to the respective substance users.

The full eligibility criteria are described in Supplement section 1. In brief, all participants were aged between 18 and 45 years. For substance user groups, inclusion criteria were having used MDMA or METH on at least 25 lifetime occasions and at least once within the last 6 or 12 months, respectively. Either MDMA or METH also had to be the main illicit substance of use. For amphetamine-naïve healthy controls, inclusion criteria were less than 15 lifetime occasions for any illicit substance use (except for cannabis), no intake of amphetamines or other stimulants within the last 4 months, no DSM-5 substance use disorder except for nicotine, mild alcohol, or cannabis use disorder, no daily cannabis use and/or daily intake of psychotropic medication for more than 5 years in childhood/adolescence. Exclusion criteria for all participants included severe somatic, neurological, or psychiatric disorders, which would have affected task performance due to likely impairments in formal thinking, severe, substance-unrelated executive dysfunction, changes in perception, or retardation of motor responses. We further excluded pregnancy or breast-feeding, as well as illicit substance intake within 48 h prior to study appointment as assessed by urine drug screening. The study was conducted in accordance with the Declaration of Helsinki and approved by the local Ethic Committees of the Canton Zurich (BASEC-Nr. 2018–02125) and the TU Dresden (EK 69022018). All participants provided written informed consent and were reimbursed for their participation.

### 2.2 Clinical assessment

Clinically trained examiners assessed all participants for common DSM-IV psychiatric disorders such as depression, anxiety or psychotic episodes with a standardized screening tool and for substance use disorders (SUDs) using the Structural Clinical Interview for DSM-5 criteria (SCID-5) (Beesdo-Baum et al., 2019). In order to avoid artificially stratifying the sample, participants were only excluded from the sample if the examiners detected a current psychiatric disorder that was deemed severe enough to interfere with task performance. Verbal intelligence was measured with a German multiple-choice vocabulary test (Lehrl et al., 1995). Given that chronic stimulant use is often associated with elevated depressive and/or ADHD symptoms, as well as increased impulsivity, all participants filled out additional questionnaires. To assess depressive symptoms in the last two weeks, we used the CESD-R (Eaton et al., 2004). General ADHD symptoms were assessed with the ADHD-SR (Rösler et al., 2004) and impulsivity was evaluated with the BIS (Patton et al., 1995). To test whether user groups showed elevated state- or trait-anger expressions, all participants filled out the STAXI-2 (Rohrmann et al., 2013).

### 2.3 Substance use assessment

History of legal and illegal substance use of all study participants was recorded by applying the Interview for Psychotropic Drug Consumption (Quednow et al., 2004). In the METH user group, current methamphetamine craving was estimated using an adaption of the Brief Cocaine Craving Questionnaire (Sussner et al., 2006). Since the majority of MDMA users do not typically report strong craving (Davis and Rosenberg, 2014), it was not assessed for the MDMA user group, nor any of the control groups. In all study groups, hair and urine samples were collected as objective measurements of chronic and acute substance use, respectively. Whenever possible, a scalp-near hair sample was taken from the back of the head. Otherwise, body hair was collected from lower arm, leg or chest. Hair analyses for a wide range of substances was performed with liquid chromatography-tandem mass spectrometry (LC-MS/MS) as detailed in Scholz et al. (2021). Recent illicit substance intake was assessed by a multi-drug urine screening (Innovacon, Inc.) for cocaine, amphetamine, methamphetamine, MDMA, cannabis, methadone, morphine, barbiturates, benzodiazepines, and buprenorphine at the beginning of the study appointment.

### 2.4 Experimental paradigm

**Fig. 1:**
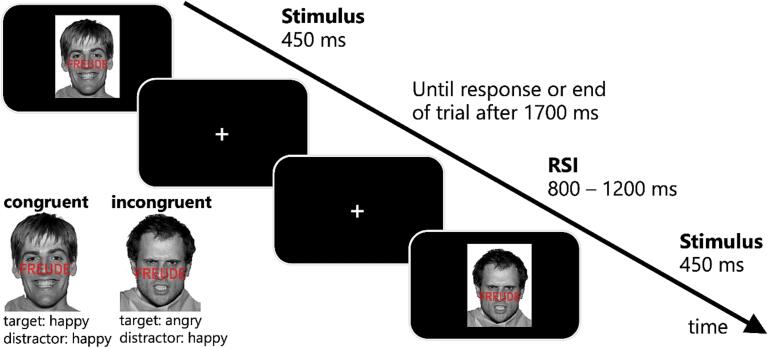
Exemplary stimuli and the time course of a trial in the emotional face-word Stroop paradigm. Participants were asked to correctly assess the facial expression (i.e., emotional target information) as happy or angry, while disregarding the German word for happiness or anger (i.e., emotional distractor information), which was written across the face. A conflict evolved in incongruent trials, in which target and distractor mismatched, while stimuli were congruent when target and distractor matched. In each trial, the stimulus was presented for 450 ms, which was followed by a centrally presented white fixation cross until the executed response or the end of the trial after 1700 ms. Between trials, a response stimulus interval (RSI) was jittered between 800 and 1200 ms presenting a central fixation cross.

We used an adapted version of the emotional face-word Stroop paradigm (Etkin et al., 2006) reported by Schreiter et al. (Schreiter et al., 2018, Schreiter et al., 2018) to investigate conflict processing of emotional information (Fig. 1).

In the paradigm, a conflict evolves between two incompatible kinds of emotional information, which are simultaneously presented in one stimulus. Each stimulus consisted of an image depicting a greyscale emotional face and an emotional word inscribed in red capital letters across the middle of the face. While the facial expression reflected the target information, the written word reflected the distractor information. Each image showed either a happy or an angry facial expression and either the German word for anger or happiness (i.e., “Ärger” or “Freude”). Depending on the congruency of the emotional word and the facial expression, four conditions emerged: i.e., congruent angry (i.e., angry face, word anger), congruent happy (i.e., happy face, word happiness), incongruent angry (i.e., angry face, word happiness), and incongruent happy (i.e., happy face, word anger). Participants were instructed to correctly classify the facial expression (emotional target information) as quickly and as accurately as possible, while disregarding the word (emotional distractor information). The experiment took approximately 20 min. Participants were encouraged to use designated breaks to avoid fatigue effects. In total, 16 different stimuli (resulting from the four described condition combinations and the faces of four different males) were presented equally often in a pseudo-randomised order. More details on the paradigm are outlined in Supplement section 2.

### 2.5 Electroencephalography (EEG) recording and analyses

Using amplifiers from Brain Products Inc. and electrode Fpz as a reference, we recorded high-density EEG data at a sampling rate of 500 Hz using 60 Ag-AgCl electrodes arranged in equidistance. Electrode impedances were kept beneath 5 kΩ. To pre-process the EEG data, we used the Automagic toolbox (Pedroni et al., 2019) and EEGLAB (Delorme and Makeig, 2004) on Matlab R2019a (The MathWorks Corp.), which is detailed in Supplement section 3. The pre-processed data was then segmented for each condition combination using Brain Vision Analyzer 2 (Brain Products Inc.). Segments were stimulus-locked and only included trials with correct responses. All segments started 2000 ms before and ended 2000 ms after stimulus onset. Frequencies above 20 Hz were further filtered from the data. To double-check for artifacts, an automated artifact rejection method was performed (rejection criteria: value difference above 200 μV in a 200 ms interval, amplitudes below −100 μV and above 100 μV, activity below 0.5 μV in a 100 ms interval). Next, we applied a current source density transformation to obtain a sharper contrast of spatial distribution of the electrical field across the scalp. Thus, relevant electrodes showing cognitive process-related activity are easier to identify (Tenke and Kayser, 2012). The data were further baseline-corrected using the time window between −200 to 0 ms before stimulus onset. Eventually, segments were averaged for each condition combination at the single-subject level. ERP components were quantified based on visual inspection and scalp topography (averaged for all groups): P1 at electrodes P7 and P8 from 100 to 130 ms, N1 at electrodes P7 and P8 from 155 to 180 ms, N2 at electrode Cz and CPz from 205 to 300 ms, P3 at electrodes P3 and P4 from 355 to 410 ms, and CSP at electrode CPz from 490 to 630 ms.

### 2.6 Statistical analyses

The behavioral data were separately analyzed for accuracy and reaction time (RT). Only trials in which participants responded correctly within 200 to 1200 ms were included in the analyses. The neurophysiological data were separately analyzed for the mean amplitude of the P1, N1, N2, P3, and CSP. Separate mixed analyses of variance (ANOVAs) were employed to analyze the behavioral and neurophysiological data. Subsample (MDMA [users + controls] sample assessed in Zürich vs. METH [users + controls] sample assessed in Dresden) and group (substituted amphetamine users [MDMA + METH] vs. controls [healthy controls of both sites]) were used as between-subject factors, while congruency (congruent vs. incongruent), valence (happy vs. angry), and electrode (wherever applicable) were used as within-subject factors. Whenever necessary, post hoc tests were Bonferroni-corrected. In case of non-significant trends (p ≤ 0.10) of main or interaction effects of the factors subsample and/or group, the Bayesian approach introduced by Masson (Masson, 2011) was conducted to examine which hypothesis (H_0_ or H_1_) was more likely, given the obtained data. The resulting posterior probability of H_i_ was categorized according to Raftery (Raftery, 1995). If not otherwise stated, the mean and standard error of the mean (SEM) are reported for descriptive statistics.

A sensitivity analysis run with GPower Software (Faul et al., 2009) indicated that a sample size of n = 200 participants (for a within-between interaction in case of 4 between-subject groups and 4 within-subject conditions; at an α of 5 % and a power of 95 %) was sufficient to detect effect sizes of f = 0.2 and larger.

## 3 Results

### 3.1 Sample characteristics

The final sample included *n* = 163 participants: 42 MDMA users (n = 12 with some form of MDMA-related SUD, as diagnosed with the SCID-5), 42 MDMA controls, 38 METH users (n = 38 with some form of METH-related SUD, as diagnosed with the SCID-5) and 41 METH controls. Demographic, questionnaire, and substance-related characteristics for each group are summarized in Table 1. For more details, please refer to Supplement section 1. Both user groups and their respective control groups are comparable regarding sex, age, and trait-anger global score. Positive urine tests were observed in both subsamples. In the MDMA [users + controls] sample, *n* = 2 controls showed a positive drug urine test for cannabis, while *n* = 1 MDMA user was positive for cannabis and cocaine. In the METH [users + controls] sample, a total of *n* = 11 users showed positive drug urine test (*n* = 9 for METH, *n* = 5 for amphetamine, *n* = 1 for MDMA, *n* = 5 for cannabis). These participants were nevertheless included in the statistical analyses. However, the effect of positive urine test was additionally analysed as reported in Supplement section 5.2. Of note, amphetamine is a major metabolite of METH and METH can potentially be adulterated with MDMA (especially during the COVID-19 pandemic, when the nightlife culture was disrupted and MDMA demand declined^1^). Given that the participant with a positive urine MDMA (+METH + amphetamine) result had neither intentionally consumed MDMA in the last 12 months nor showed MDMA hair residuals, we included the participant nevertheless.

**Table 1:** Demographic, questionnaire, and substance-related variables for each group per subsample.

	MDMA control (*n* = 42)	MDMA user (*n* = 42)	METH control (*n* = 41)	METH user (*n* = 38)	Significant group differences
Sex (female/male)	(24/18)	(22/20)	(12/29)	(12/26)	
Age in years	29.6 ± 1.0	29.9 ± 1.0	30.2 ± 1.0	29.5 ± 1.0	
School education in years	10.1 ± 0.2	10.4 ± 0.2	10.4 ± 0.1	9.6 ± 0.1	MDMA user > METH user * (t(69.875) = 2.712; p_corr_ = 0.034) METH control > METH user ** (t(77) = 3.459; p_corr_ = 0.004)
Verbal IQ	105.6 ± 1.6	102.1 ± 1.6	104.6 ± 1.3	99.0 ± 1.6	METH control > METH user * (t(77) = 2.664; p_corr_ = 0.038)
ADHD total score	10.6 ± 1.1	13.3 ± 1.5	10.0 ± 1.1	15.3 ± 1.6	METH control < METH user * (t(65.599) = 2.629; p_corr_ = 0.043)
BIS total score	62.9 ± 1.3	65.2 ± 1.5	60.5 ± 1.0	69.1 ± 1.5	METH control < METH user *** (t(76) = 4.727; p_corr_ < 0.001)
CESD total score	9.9 ± 1.2	12.0 ± 1.6	7.1 ± 0.8	18.8 ± 2.2	METH control < METH user *** (t(48.700) = 4.914; p_corr_ < 0.001)
STAXI state anger total score	16.0 ± 0.3	17.0 ± 0.9	15.1 ± 0.0	15.6 ± 0.4	
STAXI trait anger total score	17.6 ± 0.5	18.5 ± 0.9	17.8 ± 0.6	19.4 ± 0.9	
Meth craving total score	–	–	–	59.2 ± 1.9	*No statistical comparisons made*
Alcohol					
Years of use	12.1 ± 1.1	13.8 ± 1.1	13.6 ± 1.0	12.4 ± 1.1	
Cum. lifetime dose in kg	93.5 ± 21.0	158.3 ± 32.1	57.2 ± 11.5	236.9 ± 52.1	METH control < METH user ** (t(40.627) = 3.364; p_corr_ = 0.007)
Cannabis					
Years of use	4.6 ± 1.0	9.3 ± 1.1	0.6 ± 0.3	8.5 ± 1.2	METH control < METH user *** (t(43.406) = 6.248; p_corr_ < 0.001) MDMA control < MDMA user * (t(82) = 3.029; p_corr_ = 0.013) METH control < MDMA control ** (t(51.087) = 3.734; p_corr_ = 0.002)
Cum. lifetime dose in g	217.3 ± 92.5	752.8 ± 192.6	8.3 ± 5.8	2847.0 ± 938.7	METH control < METH user * (t(37.003) = 3.024; p_corr_ = 0.018)
THC hair concentration in pg/mg	0.0 ± 0.0	0.0 ± 0.0	0.0 ± 0.0	0.0 ± 0.0	METH control < METH user ** (U = 595.000; p_corr_ = 0.002) MDMA control < MDMA user *(U = 1073.000; p_corr_ = 0.020)
MDMA					
Used in last 12 months	*n = 0*	*n = 42*	*n = 0*	*n = 9*	
Weekly occasions	0.0 ± 0.0	0.3 ± 0.0	0.0 ± 0.0	0.0 ± 0.0	MDMA control < MDMA user *** (t(41.000) = 6.156; p_corr_ < 0.001) METH user < MDMA user *** (t(41.557) = 5.923; p_corr_ < 0.001)
Weekly amount in gram	0.0 ± 0.0	0.1 ± 0.0	0.0 ± 0.0	0.0 ± 0.0	MDMA control < MDMA user *** (t(41.000) = 6.234; p_corr_ < 0.001) METH user < MDMA user *** (t(41.807) = 6.027; p_corr_ < 0.001)
Years of use	0.0 ± 0.0	8.9 ± 0.8	0.0 ± 0.0	3.2 ± 0.8	MDMA control < MDMA user *** (t(41.000) = 10.268; p_corr_ < 0.001) METH user < MDMA user *** (t(78) = 4.549; p_corr_ < 0.001) METH control < METH user ** (t(37.000) = 3.679; p_corr_ = 0.003)
Cum. lifetime dose in g	0.0 ± 0.0	76.0 ± 17.1	0.0 ± 0.0	33.1 ± 9.4	MDMA control < MDMA user *** (t(41.000) = 4.436; p_corr_ < 0.001) METH control < METH user ** (t(37.000) = 3.495; p_corr_ < 0.005)
Days since last use	2774.5 ± 355.5	21.8 ± 2.7	731.0 ± 0.0	1667.0 ± 331.2	METH user > MDMA user *** (t(32.004) = 4.966; p_corr_ < 0.001
Hair concentration in pg/mg	0.0 ± 0.0	809.0 ± 557.5	0.0 ± 0.0	13.0 ± 13.0	MDMA control < MDMA user *** (U = 1721.000; p_corr_ < 0.001) METH user < MDMA user *** (U = 71.000; p_corr_ < 0.001) METH control < METH user *** (U = 770.000; p_corr_ < 0.001)
METH					
Used in last 12 months	*n = 0*	*n = 1*	*n = 0*	*n = 38*	
Weekly occasions	0.0 ± 0.0	0.0 ± 0.0	0.0 ± 0.0	3.1 ± 0.3	METH control < METH user *** (t(37.000) = 7.988; p_corr_ < 0.001) MDMA user < METH user *** (t(37.000) = 7.986; p_corr_ < 0.001)
Weekly amount in gram	0.0 ± 0.0	0.0 ± 0.0	0.0 ± 0.0	1.5 ± 0.3	METH control < METH user *** (t(37.000) = 5.232; p_corr_ < 0.001) MDMA user < METH user *** (t(37.000) = 5.232; p_corr_ < 0.001)
Years of use	0.0 ± 0.0	0.0 ± 0.0	0.0 ± 0.0	9.1 ± 0.8	METH control < METH user *** (t(37.000) = 10.656; p_corr_ < 0.001) MDMA user < METH user *** (t(37.000) = 10.656; p_corr_ < 0.001)
Cum. lifetime dose in g	0.0 ± 0.0	0.0 ± 0.0	0.0 ± 0.0	1438.2 ± 287.5	METH control < METH user *** (t(37.000) = 5.002; p_corr_ < 0.001) MDMA user < METH user *** (t(37.000) = 5.002; p_corr_ < 0.001)
Days since last use	–	222.0 ± 0.0	–	69.7 ± 16.0	*[only one data point in the MDMA user group]*
Hair concentration in pg/mg	0.0 ± 0.0	0.0 ± 0.0	0.0 ± 0.0	905.0 ± 881.5	METH control < METH user ** (U = 908.000; p_corr_ = 0.002) MDMA user < METH user *** (U = 1077.000; p_corr_ < 0.001)

*Note*: Substance use in both weekly occasions and weekly amount in gram refer to the last 12 months. Hair data was collected for *n* = 41 MDMA controls, *n* = 42 MDMA users, *n* = 35 METH controls and *n* = 26 METH users. Because of a strongly positive skewness, values of hair concentrations are given as median ± median absolute deviation (all other values are given as mean ± standard error of the mean). Total scores of ADHD, BIS, and CESD have data missing for one METH control. Total scores of STAXI state and trait anger have data missing for three MDMA users. As none of the control participants in either group reported to have ever consumed METH, there is no control group data for days since last (METH) use. *ADHD* = Attention-Deficit/Hyperactivity Disorder; *BIS* = Barratt Impulsiveness Scale; *CESD* = Center for Epidemiologic Studies Depression Scale; *STAXI* = State-Trait Anger Expression Inventory; *THC* = tetrahydrocannabinol; * = p <.05; ** = p <.01; *** = p <.001 (all p values were Bonferroni-corrected depending on the number of group comparisons run for the respective variable).

### 3.2 Behavioral data

**Fig. 2:**
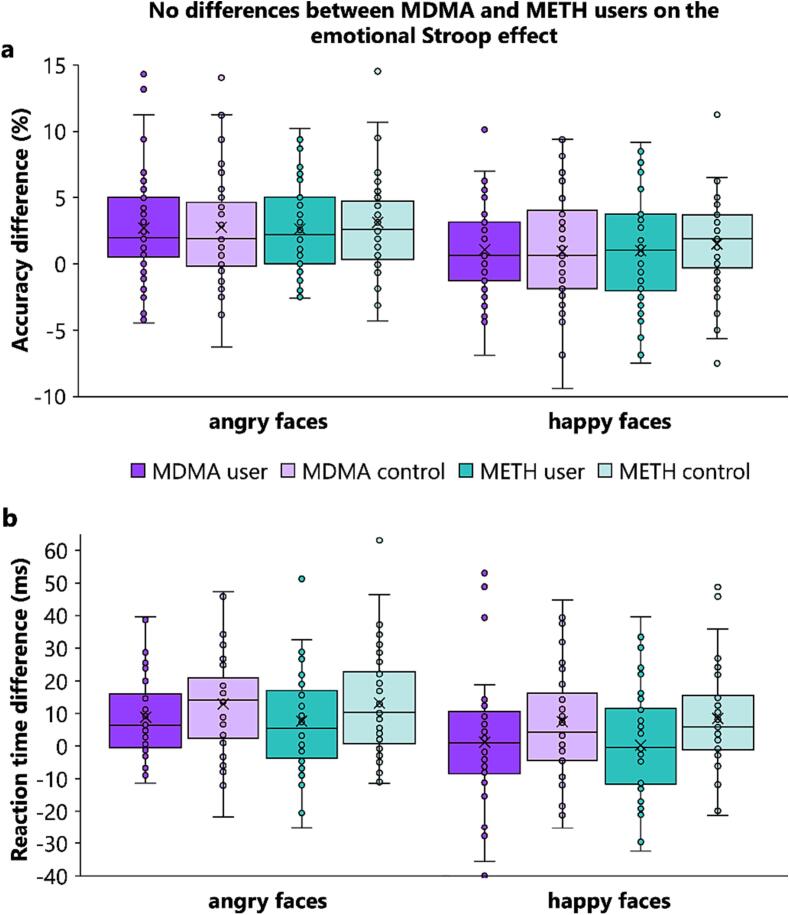
Illustration of the Stroop effects: (**a**) the performance difference of congruent minus incongruent trials for accuracy in percent, and (**b**) incongruent minus congruent trials for reaction time in milliseconds. As depicted, the emotional Stroop effects were determined for each valence condition (angry vs. happy) and each subsample x group combination (i.e., MDMA users, MDMA controls, METH users, and METH controls). For both measures, there were no interaction effects between subsample and group (with congruency and/or valence). Consequently and against our hypothesis, METH users did **not** show stronger Stroop effects in angry condition, as compared to MDMA users. In the happy condition, however, we expected and also found no differences between METH and MDMA users in the Stroop effect.

**Fig. 3:**
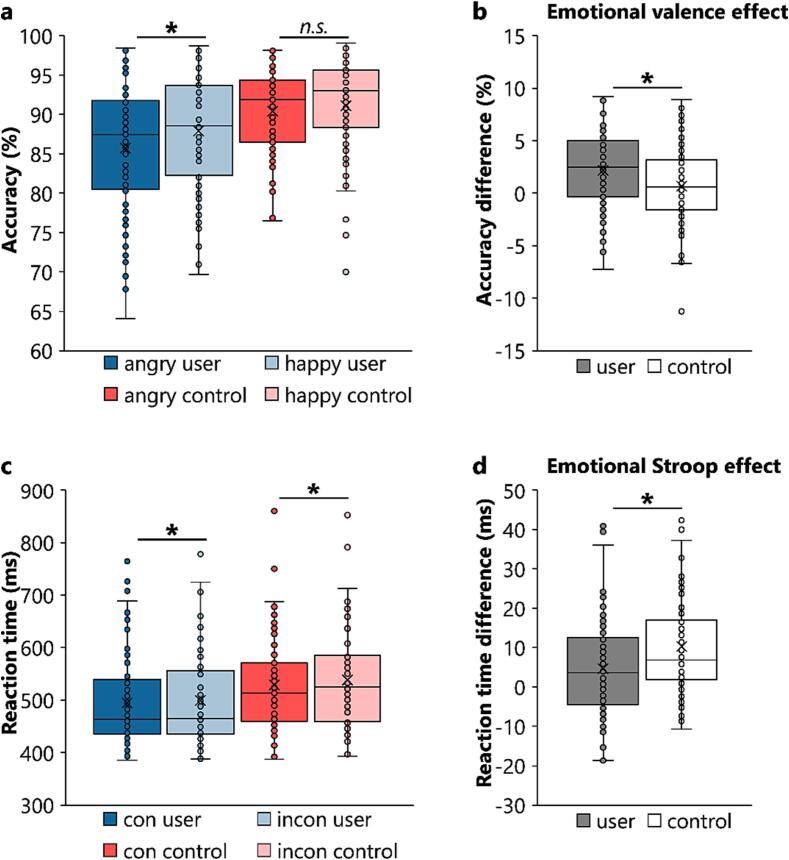
Illustration of the behavioral interaction effects between group and emotional valence found for accuracy (in percent, upper graphs) and between group and congruency found for reaction time (RT in milliseconds, lower graphs). (**a**) Users responded significantly less accurately in angry than in happy trials, while controls did not. (**b**) Thus, the size of the emotional valence effect (i.e., happy minus angry trials) was significantly larger for users relative to controls. (**c**) Both users and controls responded significantly faster in congruent (con) than in incongruent (incon) trials. (**d**) Yet, the size of the emotional Stroop effect (i.e., incongruent minus congruent) was significantly smaller for users relative to controls. Please note, that for both measures, there were no interaction effects between subsample and group (either on its own, or with congruency and/or valence), indicating that MDMA and METH users did not significantly differ in this task.

In terms of the task-related data (i.e., congruency and valence without group effects), we found the same task effects as previously reported (Schreiter et al., 2018, Schreiter et al., 2018, Schreiter et al., 2019). They are detailed in Supplement section 5.1. Substance-related effects are shown in Fig. 2, Fig. 3 and detailed below.

Contrary to expectations, the mixed ANOVA for accuracy revealed no interaction effects between subsample and group (all *F* ≤ 2.862; *p* ≥ 0.093) indicating no significant differences between MDMA and METH users. Additional Bayesian analysis for the non-significant trend interaction between group and subsample (*F* = 2.862; *p* =.093) revealed positive evidence in favour of the H_0_ (*p*(H_0_|D) = 92,7 %). We thus did not perform exploratory post-hoc tests. Nevertheless, a main effect of subsample (*F_(1,159)_* = 4.374; *p* =.038; *η_p_^2^* = 0.027) showed higher accuracy in the MDMA [users + controls] sample (89.7 % ± 0.6) than in the METH [users + controls] sample (87.7 % ± 0.7), while a main effect of group (*F_(1,159)_* = 16.955; *p* <.001; *η_p_^2^* = 0.096) revealed that substituted amphetamine users (86.7 % ± 0.6) generally responded less accurately than the controls (90.7 % ± 0.6). Interestingly, the mixed ANOVA yielded a significant interaction between group and valence (*F_(1,159)_* = 4.649; *p* =.033; *η_p_^2^* = 0.028). Separate post hoc paired-samples t-tests for each group showed that substituted amphetamine users’ accuracy in the angry condition (85.7 % ± 0.9) was lower than in the happy condition (87.9 % ± 0.7) (*t_(79)_* = -3.813; *p* <.001; *d* = -0.426). No such difference was evident in controls (*t_(82)_* = -1.677; *p* =.194). The valence effect (i.e., happy minus angry condition), was larger in substituted amphetamine users (|2.1| % ± 0.5) than in controls (|0.6| % ± 0.4) (*t_(1 6 1)_* = -2.185; *p* =.030; *d* = -0.342). No further interaction effects involving the group factors reached significance for the accuracy data (all *F* ≤ 0.357; *p* ≥ 0.551).

In the mixed ANOVA for RTs, no interaction effects between subsample and group were found (all *F* ≤ 0.875; *p* ≥ 0.351), implying no significant differences between MDMA and METH users. Instead, we found a main effect of group (*F_(1,159)_* = 6.364; *p* =.013; *η_p_^2^* = 0.038), with faster correct responses in substituted amphetamine users (497 ms ± 10.1) than in controls (533 ms ± 9.9). More importantly, there was a significant interaction between congruency and group (*F_(1,159)_* = 7.775; *p* =.006; *η_p_^2^* = 0.047). Separate post hoc paired-samples t-tests for each group showed that congruent trials yielded faster responses than incongruent trials in both the controls (*t_(82)_* = -7.140; *p* <.001; *d* = -0.784) and the substituted amphetamine users (*t_(79)_* = -3.329; *p* =.002 *d* = -0.372). Yet, the Stroop effect (i.e., incongruent minus congruent) was smaller in substituted amphetamine users (4 ms ± 1.4) than in controls (10 ms ± 1.4) (*t_(1 6 1)_* = 2.787; *p* =.006; *d* = 0.437). No other main or interaction effects involving the group factors reached significance for the RT data (all *F* ≤ 2.239; *p* ≥ 0.137).

Potential confounders of the observed substance-specific effects in the overall sample, as well as associations between behavioural measures and MDMA- and METH-specific variables in the user groups are further explored in Supplement sections 5.2 and 5.3. In brief, the interaction between group and valence was no longer significant for accuracy when controlling for depressive symptoms (*F*_(1,157)_ = 2.499; *p* =.116). Substance-specific correlates were only evident for MDMA users: i) Users with higher levels of MDMA concentration in hair performed the task more slowly, and ii) the more MDMA was used per week over the past 12 months, the slower MDMA users responded in a) happy and b) congruent trials.

### 3.3 Neurophysiological data

**Fig. 4:**
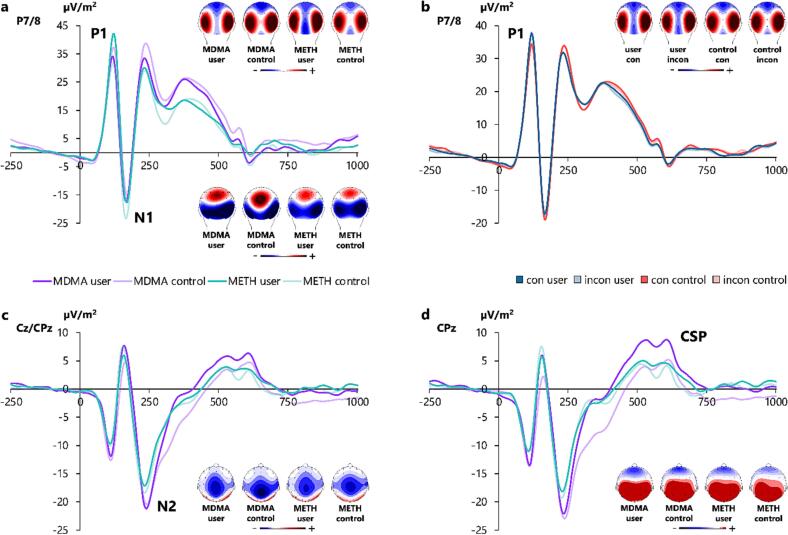
Illustration of ERP components for each subsample x group combination (MDMA user, MDMA control, METH user, METH control in graph a, c, d) and P1 amplitudes for each group x congruency combination in terms of the three-way interaction effect between congruency, group, and electrode (in graph b). (**a**) P1 (100 – 130 ms) and N1 (155 – 180 ms), averaged over electrodes P7 and P8. (**b**) P1 (100 – 130 ms), averaged over electrodes P7 and P8. Post-hoc analyses, however, did not reveal significant group differences in congruent (con) or incongruent (incon) trials. (**c**) N2 (205 – 300 ms) averaged over electrodes Cz and CPz. (**d**) Conflict slow potential (CSP, 490 – 630 ms) at electrode CPz. In all graphs, time point zero marks the stimulus onset, and maps of scalp topography depict the electrical potential in the respective time windows of each ERP component. In the topographies, positivity is marked in red, while negativity is marked in blue. (For interpretation of the references to colour in this figure legend, the reader is referred to the web version of this article.)

**Fig. 5:**
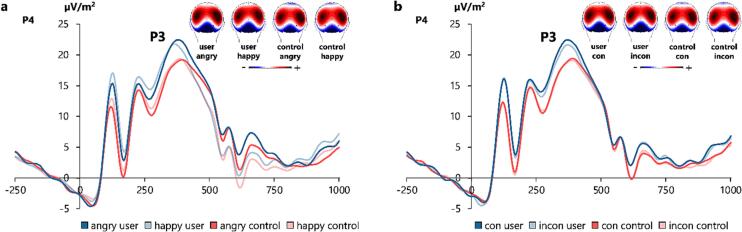
Illustration of P3 ERP amplitudes (355 – 410 ms, at electrode P4) for (**a**) the significant interaction effect between emotional valence, group, and electrode, and (**b**) the non-significant trend interaction between congruency, group, and electrode. (**a**) Users exhibited larger P3 ERP amplitudes in angry than in happy trials at electrode P4, whereas controls did not. (**b**) Users yielded larger P3 ERP amplitudes in congruent (con) than in incongruent (incon) trials at electrode P4, whereas controls did not. In both graphs, time point zero marks the stimulus onset, and maps of scalp topography depict the electrical potential in the time window of the P3 ERP amplitude. In the topographies, positivity is marked in red, while negativity is marked in blue. (For interpretation of the references to colour in this figure legend, the reader is referred to the web version of this article.)

The P1, N1, N2, and CSP are illustrated in Fig. 4, while Fig. 5 shows the P3. To focus this section, we only report significant ERP effects that reflect the (pattern of the) observed behavioural effects, as ERP effects that cannot be found in the behavioural data most likely reflect mere epiphenomena. Task effects are reported in Supplement section 6.

For the P1 amplitude at electrodes P7/P8, the mixed ANOVA revealed a significant three-way interaction between group, congruency and electrode (*F_(1,159)_* = 5.365; *p* =.022; *η_p_^2^* = 0.033). Separate post-hoc repeated-measures ANOVAs for each group showed a significant interaction between congruency and electrode in substituted amphetamine users (*F_(1,79)_* = 6.815; *p* =.022; *η_p_^2^* = 0.079), but not in controls (*F_(1,82)_* = 0.110; *p* = 1.0). A post-hoc independent sample *t*-test showed that in the substituted amphetamine user group, the P1 Stroop effect (incongruent minus congruent) was larger at electrode P8 (|0.6| μV/m^2^ ± 0.4) than at electrode P7 (|0.4| μV/m^2^ ± 0.3) (*t_(79)_* = -2.611; *p* =.011; *d* = -0.292). No other main or interaction effects corresponding to the behavioural results involving the group factors emerged (all *F* ≤ 1.010; *p* ≥ 0.316).

For the N1 amplitudes at electrodes P7/P8, we found a non-significant trend for an interaction between subsample and electrode (*F_(1,159)_* = 3.417; *p* =.066; *η_p_^2^* = 0.021). Because additional Bayesian analysis showed rather weak evidence in favour of the H_0_ for this effect (*p*(H_0_|D) = 64.5 %), we refrained from exploratory post-hoc analyses. Furthermore, a non-significant trend was observed for the interaction between group and valence (*F_(1,159)_* = 3.498; *p* =.063; *η_p_^2^* = 0.022). Add-on Bayesian analysis also revealed more evidence in favour of the H_0_ for this effect (*p*(H_0_|D) = 68,4 %). We thus did not perform exploratory post-hoc tests. No other main or interaction effects corresponding to the behavioural results involving the group factors were obtained (all *F* ≤ 3.498; *p* ≥ 0.063).

For the N2 at electrodes Cz and CPz, we obtained a main effect of subsample (*F_(1,159)_* = 4.320; *p* =.039; *η_p_^2^* = 0.026), with smaller N2 amplitudes in the METH [users + controls] sample (-13.5 μV/m^2^ ± 0.8) than in the MDMA [users + controls] sample (-16.0 μV/m^2^ ± 0.8). No other main or interaction effects corresponding to the behavioural results involving the group factors were observed (all *F* ≤ 2.024; *p* ≥ 0.157).

For the CSP amplitudes at electrode CPz, the mixed ANOVA revealed a significant main effect of subsample (*F_(1,159)_* = 8.206; *p* =.005; *η_p_^2^* = 0.049), showing a smaller CSP in the METH [users + controls] sample (3.9 μV/m^2^ ± 0.5) than in the MDMA [users + controls] sample (6.0 μV/m^2^ ± 0.5). Furthermore, a main effect of group (*F_(1,159)_* = 12.835; *p* <.001; *η_p_^2^* = 0.075) indicated a larger CSP in substituted amphetamine users (6.2 μV/m^2^ ± 0.5) than in controls (3.6 μV/m^2^ ± 0.5). No other main or interaction effects corresponding to the behavioural results involving the group factors were obtained (all *F* ≤ 0.885; *p* ≥ 0.348).

For the P3 amplitudes at electrodes P3/P4, we found a significant three-way interaction between group, valence, and electrode (*F_(1,159)_* = 5.626; *p* =.019; *η_p_^2^* = 0.034). To resolve this interaction, we ran separate post-hoc mixed ANOVAs for each electrode. The interaction between group and valence was observed at electrode P4 (*F_(1,161)_* = 4.572; *p* =.034; *η_p_^2^* = 0.028), but not at electrode P3 (*F_(1,161)_* = 1.003; *p* =.318). Further post hoc paired-samples t-tests at electrode P4 yielded larger amplitudes in the angry (22.1 μV/m^2^ ± 1.2) than in the happy condition (21.1 μV/m^2^ ± 1.1) in users (*t_(79)_* = 2.326; *p* =.046; *d* = 0.260), whereas control participants did not differ in this way (*t_(82)_* = -0.468; *p* = 1.0). Consequently, the valence effect (angry minus happy) at electrode P4 was larger in substituted amphetamine users (|1.0| μV/m^2^ ± 0.4) than in controls (|0.1| μV/m^2^ ± 0.3) (*t_(1 6 1)_* = -2.138; *p* =.034; *d* = -0.335). Furthermore, we found a non-significant trend for a three-way interaction between group, congruency and electrode (*F_(1,159)_* = 3.257; *p* =.073; *η_p_^2^* = 0.020). Additional Bayesian analysis revealed positive evidence in favour of the H_1_ (*p*(H_1_|D) = 86.5 %). We thus ran exploratory post-hoc ANOVAs for each electrode site. The interaction between group and congruency was at trend level at electrode P4 (*F_(1,161)_* = 3.164; *p* =.077; *η_p_^2^* = 0.019), but not at electrode P3 (*F_(1,161)_* = 0.096; *p* =.758). Further post hoc paired-samples t-tests for each group at electrode P4 showed that substituted amphetamine users yielded larger P3 amplitudes in the congruent (22.0 μV/m^2^ ± 1.2) than in the incongruent condition (21.2 μV/m^2^ ± 1.1) (*t_(79)_* = 3.207; *p* =.004 *d* = 0.359), whereas controls did not (*t_(82)_* = 1.111; *p* =.540). The Stroop effect (incongruent minus congruent) at electrode P4 showed a trend towards being slightly larger in substituted amphetamine users (|0.8| μV/m^2^ ± 0.2) than in controls (|0.2| μV/m^2^ ± 0.2) (*t_(1 6 1)_* = 1.779; *p* =.077). No other main or interaction effects corresponding to the behavioural results involving the group factors emerged (all *F* ≤ 2.933; *p* ≥ 0.089).

## 4 Discussion

Previous work has shown that both chronic MDMA and chronic METH users display deficits in impulse control functions (Fernández-Serrano et al., 2011, Potvin et al., 2018, Lee et al., 2019); which may impair social interactions of the affected individuals. However, poor social-cognitive functions (Henry et al., 2009, Homer et al., 2008, Uhlmann et al., 2018) and aggressive tendencies (Payer et al., 2011, Kim et al., 2011) have been related more to chronic METH use, rather than to chronic MDMA use (Carlyle et al., 2019, Wunderli et al., 2017, Hoshi et al., 2007). In light of this, we investigated the effects of chronic MDMA and METH use on conflict monitoring processes relative to different social-affective information (i.e., anger and happiness), and their underlying neurophysiological mechanisms using an emotional Stroop paradigm. We expected that conflict control in angry (but not happy) contexts would be more impaired in METH than in MDMA users because impulsive behaviour is strongly related to aggression (Brennan and Baskin-Sommers, 2019, Ramírez and Andreu, 2006), because the catecholaminergic system (i.e., dopamine and noradrenaline) is strongly linked to inhibitory control (Robbins and Arnsten, 2009, Yu et al., 2022, Beste et al., 2016), and because dopamine is more strongly affected after chronic METH intake than after chronic MDMA intake (Roberts et al., 2016, Ashok et al., 2017).

Contrary to our hypothesis, chronic METH and MDMA users did not differ in their conflict monitoring processes, in particular not in terms of angry stimuli. Instead, we found a smaller emotional Stroop effect in substituted amphetamine users (i.e., indiscriminately in both MDMA and METH users), which seemed to be independent from emotional valence. The smaller emotional Stroop effect indicates a smaller modulatory effect of cognitive-emotional conflict control onto behavior in chronic substituted amphetamines users than in healthy controls. This contrasts with findings from classical conflict tasks (e.g., flanker, stroop, simon tasks), the majority of which showed inhibitory control deficits among METH and MDMA users, as compared to controls (Potvin et al., 2018, Lee et al., 2019, Smith et al., 2014). A major difference between classical and emotional Stroop tasks lies in the stimulus material used. In this emotional Stroop task, participants responded to emotional faces, while ignoring emotional words. However, face recognition is an inherent ability of both humans and animals (Gross et al., 1972), while visual word recognition is an acquired ability (Takamiya et al., 2020). Therefore, faces could be more likely to draw our attention than words. As faces were task-relevant, this could have led to reduced conflict effects in general. Further given that task-relevant faces might have been more salient than task-irrelevant words, the Stroop conflict may have been less strongly perceived/detected by substituted amphetamine users relative to controls in this particular task. In future studies, we hence suggest implementing emotional Stroop tasks using emotional words as target stimuli and facial expressions as distractor stimuli to potentially induce stronger conflicts.

As both METH and MDMA users did not differ in cognitive-emotional conflict control, we suggest that neither the expected METH-related dopaminergic downregulation (Ashok et al., 2017) nor the MDMA-related changes in serotonin function (Roberts et al., 2016) can sufficiently explain the observed reduction of conflict effects. Moreover, elevated serotonin levels have been associated with stronger control over social behaviour (Steenbergen et al., 2016). Interestingly, both MDMA and METH are known to acutely increase noradrenaline release (Hysek et al., 2011, Gouzoulis-Mayfrank and Daumann, 2009). Noradrenaline plays a crucial role in conflict processing (Yu et al., 2022), in the interaction between emotion and cognition (Pessoa, 2015), and for social decision-making (Terbeck et al., 2016). Thus, noradrenaline could be a promising (additional) mechanism underlying cognitive-emotional conflict control in both user groups, although findings on noradrenergic modulations after chronic use of substituted amphetamines are still lacking. Furthermore, we had initially assumed that aggressive tendencies in METH users might contribute to more impaired cognitive-emotional conflict control. However, the groups did not differ in their self-reported anger trait expression, which may additionally preclude substance-specific emotional-cognitive conflict effects.

Importantly, the interaction effect of group and congruency was most clearly demonstrated for the P3 ERP component. Although we obtained a non-significant trend, Bayesian analysis indicated positive evidence for stronger P3 modulations between emotionally congruent and incongruent conditions in users (independently of substance group) relative to controls. The P3 ERP may reflect processes of stimulus–response (S-R) mapping (Ullsperger et al., 2014). Interestingly, catecholamines (i.e., dopamine, noradrenaline) modulate S-R mapping (Yu et al., 2022, Bensmann et al., 2020). Given the likely tolerance-induced catecholaminergic dysfunctions in substituted amphetamine users (although conclusive evidence regarding chronic changes of the noradrenaline system in these users is still lacking), S-R mapping processes may be altered after chronic use of substituted amphetamines. Thus, stronger substances-related P3 modulations may mirror enhanced engagement of cognitive resources during S-R mapping processes, which may have facilitated better performance as seen in smaller emotional Stroop effects.

We furthermore showed that both chronic MDMA and METH use impaired emotional processing of angry information, but not happy information. This is supported by Uhlmann et al., who demonstrated that chronic METH use was specifically associated with deficits in recognition of anger (Uhlmann et al., 2018). Likewise, deficits in anger recognition have been found in chronic cocaine users (Fernández-Serrano et al., 2010), who are also known to exhibit dopaminergic dysfunctions similar to those of METH users (Ashok et al., 2017). Despite lacking evidence of altered emotional processing in chronic MDMA users, serotonin depletion has been linked to increased amygdala activity in response to angry faces, suggesting that reduced serotonergic neurotransmission affects the processing of anger (Grady et al., 2013).

Importantly, an interaction effect between group and valence was once more observed within the P3 ERP time window. Our findings show larger P3 amplitudes to angry than to happy faces in users relative to controls. This matches previous evidence showing stronger P3 modulations for negative/aversive compared to positive/non-aversive emotional content (Schreiter et al., 2018, Kaestner and Polich, 2011) and may reflect increased processing capacity requirements of negative emotional content (Schreiter et al., 2018). Thus, it seems that substituted amphetamine users may require more processing resources for the categorization of and response to angry faces than happy faces. Substituted amphetamine user responses may indicate an increased sensitivity to aversive content, relative to controls. This is supported by a study in addicted METH users demonstrating a larger P3 amplitude in response to METH-related (compared to neutral) words in an addiction Stroop task (Haifeng et al., 2015).

In our study, both user groups were heterogeneous to some extent and we allowed for healthy control participants to have had low-grade recreational exposure to some drugs of abuse (as cannabis) also taken by the also taken by the participants in the user groups (in line with previous studies, e.g., Casey and Cservenka, 2020, Vonmoos et al., 2013). While we consider this a strength due to the more realistic reflection of the actual user/healthy populations, this could also have led to confounds and future studies might want to consider completely substance-naïve control participants. Nevertheless, descriptive data showed strong differences between user and control groups with respect to the relevant stimulant substances and we investigated a wide range of potential confounding variables (see Supplement), showing no influences on substance-related interaction effects (except for depression scores). Nevertheless, it is important to consider the possibility that all confounders combined might have had an aggregated effect on the results. Of note, the fact that the MDMA and METH user groups did not significantly differ from each other (as would have been evidenced by an interaction of the group and subsample factors), suggests that neither the differences in the primary substance of use, nor in their addictiveness or consumption patterns played a major role for the observed results. Furthermore, most participants in the user groups were active users who only discontinued their substance use for study participation. Thus, post-acute intoxication effects, i.e., the interaction of tolerance and (active) use, might have obscured the chronic substance effects. Specifically, catecholamine levels might differ between short-term and long-term abstinence, so that it is somewhat unclear if the same results could be obtained in a sample of users that have been abstinent for longer periods of time. Also, it should be noted that while the distinction between users with and without an SUD was not the research goal of this study, all of the users in the METH group, but only a minority of users in the MDMA group fulfilled the respective diagnostic criteria (as was to be expected; see introduction). For this reason, we refrained from additional analyses investigating this factor (even though we can of course not exclude the possibility of this factor also contributing to the variety within and between samples). Lastly, there is a need for future studies to shed further light on the potential (non–)linear relationship between behavioural and neurophysiological changes induced by prolonged stimulant (ab)use.

## 5 Conclusions

In conclusion, this is the first study comparing the effects of chronic MDMA and chronic METH use on cognitive-emotional conflict control and their neurophysiological correlates. Although our findings suggest that chronic MDMA and METH users do not differ from each other in cognitive-emotional conflict monitoring functions or in emotional processing, both MDMA and METH users exhibited differences relative to controls. Specifically, we found a smaller modulation of behavioral performance by cognitive-emotional conflict control and selectively increased deficits in emotional processing limited to anger. Both behavioral effects were underpinned by stronger P3 ERP modulations reflecting stimulus–response mapping and decision-making processes. Thus, the P3 component is an important indicator of substance-related alterations that is sensitive to different determinants (i.e., emotional valence and congruence). Yet, the underlying neurobiological mechanisms, particularly the (potentially combined) role of monoamines on cognitive-emotional conflict control in chronic MDMA and METH users, remains inconclusive.

## Role of the funding source

The funding parties had no role in study design; in the collection, analysis and interpretation of data; in the writing of the report; and in the decision to submit the article for publication.

## Funding

This international collaborative study was supported by grants from the German Research Foundation (Deutsche Forschungsgemeinschaft, DFG) TRR 265 B07 to AS and CB, BE4045/34–1 to CB and from the Swiss National Science Foundation (Schweizerischer Nationalfonds, SNF) 320030L_179450 to BBQ.

## CRediT authorship contribution statement

**Antje Opitz:** Writing – review & editing, Writing – original draft, Visualization, Investigation, Formal analysis, Data curation. **Josua Zimmermann:** Writing – review & editing, Investigation, Data curation. **David M. Cole:** Investigation. **Rebecca C. Coray:** Writing – review & editing, Data curation. **Anna Zachäi:** Investigation, Formal analysis, Data curation. **Markus R. Baumgartner:** Formal analysis. **Andrea E. Steuer:** Formal analysis. **Maximilian Pilhatsch:** Writing – review & editing, Funding acquisition. **Boris B. Quednow:** Writing – review & editing, Funding acquisition, Conceptualization. **Christian Beste:** Writing – review & editing, Funding acquisition, Formal analysis, Conceptualization. **Ann-Kathrin Stock:** Writing – review & editing, Formal analysis, Conceptualization.

## Declaration of competing interest

The authors declare that they have no known competing financial interests or personal relationships that could have appeared to influence the work reported in this paper.
