# Supplementary material for: Conflict monitoring and emotional processing in 3,4-methylenedioxymethamphetamine (MDMA) and methamphetamine users – A comparative neurophysiological study
Source: Neuroimage Clin. 2024 Feb 15;41:103579. doi: 10.1016/j.nicl.2024.103579 (PMC10924209; doi:10.1016/j.nicl.2024.103579)
Supplement: Supplementary data 1 [file mmc1.pdf]

SUPPLEMENTARY INFORMATION

Conflict monitoring and emotional processing in 3,4-methylenedioxymethamphetamine (MDMA) and methamphetamine users – a comparative neurophysiological study

Authors

Antje Opitz[1], Josua Zimmermann[2,3], David M. Cole[2,4], Rebecca C. Coray[2,3], Anna Zachäi[1], Markus R. Baumgartner[5], Andrea E. Steuer[6], Maximilian Pilhatsch[7,8], Boris B. Quednow[2,3,+], Christian Beste[1,+], Ann-Kathrin Stock[1,9,+,#]
+ Shared senior authorship

Affiliations

[1]Cognitive Neurophysiology, Department of Child and Adolescent Psychiatry, Faculty of Medicine, TU Dresden, Germany.
[2]Experimental and Clinical Pharmacopsychology, Department of Adult Psychiatry and Psychotherapy, Psychiatric University Hospital Zurich, University of Zurich, Switzerland.
[3]Neuroscience Center Zurich, University of Zurich and ETH Zurich, Switzerland.
[4]Translational Psychiatry Lab, University Psychiatric Clinics Basel, University of Basel, Basel, Switzerland
[5]Center for Forensic Hair Analytics, Institute of Forensic Medicine, University of Zurich, Zurich, Switzerland.
[6]Department of Forensic Pharmacology & Toxicology, Zurich Institute of Forensic Medicine, University of Zurich, 8057 Zurich, Switzerland
[7]Department of Psychiatry and Psychotherapy, Carl Gustav Carus University Hospital, TU Dresden, Dresden, Germany.
[8]Department of Psychiatry and Psychotherapy, Elblandklinikum, Radebeul, Germany.
[9]Biopsychology, Department of Psychology, School of Science, TU Dresden, Germany.

Table of Contents

Section 1: Sample description and eligibility criteria ---------- 2

1.1. MDMA sample ---------- 2

1.2. METH sample ---------- 3

Section 2: Experimental paradigm ---------- 3

Section 3: Pre-processing of the EEG data ---------- 4

Section 4: Sample characteristics ---------- 4

Section 5: Behavioural data ---------- 9

5.1. Task effects ---------- 9

5.2. Potential confounders in the overall sample on the observed behavioural drug effects ---------- 10

5.3. Potential confounders in MDMA and METH users on the observed behavioural and neurophysiological drug effects ---------- 11

Section 6: Task effects reflected in neurophysiological data ---------- 13

Section 1: Sample description and eligibility criteria

We initially recruited *n* = 203 volunteers (*n* = 102 users, *n* = 101 controls) as described in the main manuscript.

General exclusion criteria included a) severe somatic or neurological disorders, b) severe psychiatric disorders, which would be expected to have a negative effect on task performance, c) pregnancy or breast-feeding, d) current intake of psychotropic medication, especially substances affecting/altering monoaminergic signaling (not applicable in the METH user group due to a typically higher prescription rate of antidepressants), e) daily cannabis use (not applicable in the METH user group), f) illicit drug intake within four days prior to study appointment as measured by a urine drug screening (in case of daily METH use, abstinence was required for at least 48 hours). Given that the urine drug screening may lead to a positive result when a drug was used within four days, a positive result for methamphetamine or amphetamine was tolerated in the METH user group. Furthermore, a positive result for cannabis was tolerated in all groups because regular cannabis use can lead to longer detection times, even beyond four days prior to testing day [1]. Additional exclusion criteria for healthy controls in both samples were a) more than 15 lifetime occasions for any illicit drug use except cannabis, b) intake of ATS within the last 4 months prior to their study appointment confirmed by hair analysis, c) any substance use disorder except mild alcohol or cannabis use disorder, d) daily cannabis use in adolescence or daily intake of psychotropic medication for more than 5 years in childhood/adolescence. Additional sample-specific inclusion criteria are detailed below.

1.1. MDMA sample

Inclusion criteria for the MDMA user group were having used MDMA on at least 25 lifetime occasions and at least once within 6 months prior to study appointment. MDMA was considered to be the main illicit drug of use (except cannabis). However given that polystimulant use, combining MDMA use with cocaine or amphetamine use occasions, is quite common in this user population [2], we did not exclude polystimulant use as verified by the hair toxicology. This applied to *n* = 17 users in the final sample.

From the initially recruited MDMA sample (*n* = 51 users, *n* = 49 controls), the following exclusions were made: *n* = 1 participant dropped out of the experimental paradigm, *n* = 2 users had been using MDMA on less than 25 lifetime occasions, *n* = 4 users showed no hair residuals for MDMA, *n* = 7 controls showed hair residuals for METH or MDMA (in combination with further substances), that were most likely not reflecting external contamination, *n* = 1 participant performed the experimental paradigm close to chance level (i.e., accuracy below 60 %) in at least one task condition, and data from *n* = 1 participant represented an extreme outlier (i.e., above 3 interquartile ranges) in at least one task condition in any behavioral measure. Finally, data of *n* = 42 MDMA users and *n* = 42 matched MDMA controls entered statistical analyses. Based on the SKID interview, n = 6 of the MDMA users remaining

in the sample received a diagnosis of mild substance use disorder for MDMA, n = 5 received a diagnosis of moderate substance use disorder for MDMA, and n = 0 received a diagnosis of severe substance use disorder for MDMA.

1.2. METH sample

Inclusion criteria for the METH user group were having used METH on at least 25 lifetime occasions and at least once within 12 months prior to study appointment. METH was considered to be the main illicit drug of use (except cannabis). Given that daily METH use can be accompanied by daily alcohol or cannabis use, daily cannabis use was no reason for exclusion. This applied to *n* = 3 users in the final sample. Furthermore, METH use disorder often occurs with comorbid psychiatric disorders, such as ADHD, mood and anxiety disorder, which are often treated with psychotropic medication [3,4]. Current intake of psychotropic medication was thus tolerated in the METH user group. In the final sample, psychotropic medication was taken by *n* = 6 users and comorbid psychiatric disorders were confirmed in *n* = 14 users.

From the initially recruited METH sample (*n* = 51 users, *n* = 52 controls), the following exclusions were made: *n* = 4 participants dropped out of the study, *n* = 2 participants were pregnant or breast-feeding, *n* = 1 participant reported inconsistent data, *n* = 2 users were abstinent from METH for less than 48 hours or more than 12 months, *n* = 1 user was alcohol-intoxicated at a study appointment (breath alcohol level of 0.41 permille), *n* = 1 user showed no hair residuals for METH, *n* = 5 controls showed hair residuals for METH or MDMA, that were most likely not reflecting external contamination, *n* = 1 control had been using LSD on more than 15 lifetime occasions, *n* = 1 control had been using monoaminergic medications for more than ten years during adolescence, *n* = 1 control was on daily intake of an antidepressant medication, *n* = 3 participants performed the experimental paradigm close to chance level (i.e., accuracy below 60 %) in at least one task condition, and *n* = 2 participants were recognized as extreme outliers (i.e., above 3 interquartile ranges) in at least one task condition in any behavioral measure. Finally, data of *n* = 38 METH users and *n* = 41 matched METH controls were considered for statistical analyses. Based on the SKID interview, n = 6 of the METH users remaining in the sample received a diagnosis of mild substance use disorder for METH, n = 6 received a diagnosis of moderate substance use disorder for METH, and n = 26 received a diagnosis of severe substance use disorder for METH.

Section 2: Experimental paradigm

We used an adapted version of the emotional face-word Stroop paradigm [5] reported by Schreiter et al. [6,7] to investigate conflict processing of emotional information. We programmed the paradigm using

Presentation® software (Version 20.1, Neurobehavioral Systems, Inc., Berkeley, CA, www.neurobs.com). The images of happy and angry facial expressions of four randomly selected Caucasian men were taken from the NimStim set of facial expressions [8]. For stimuli displaying an angry facial expression, participants were required to press the left Ctrl key (on a QWERTZ keyboard) with their left index finger. For stimuli displaying a happy facial expression, the right Ctrl key had to be pressed with their right index finger. Prior to the main experiment, each participant performed a practice block of 20 trials and feedback was given after each practice trial. The main experiment consisted of four blocks of 160 trials each. Between blocks, participants were encouraged to use the designated breaks to avoid fatigue effects. In each block, each condition combination as well as all stimuli (i.e., 16 different stimuli) were presented equally often in a pseudo-randomised order. In each trial, the stimulus was presented for 450 ms against a black background, which was followed by a centrally presented white fixation cross until the response was executed or the trial ended after 1700ms. Between trials, a response stimulus interval (RSI) presenting a central fixation cross was jittered between 800 and 1200 ms.

Section 3: Pre-processing of the EEG data

To pre-process the EEG data, we used Automagic [9] and EEGLAB [10] on Matlab R2019a (The MathWorks Corp.). After down-sampling to 256 Hz and eliminating flat channels, the EEG data were re-referenced to an average reference. Next, the PREP preprocessing [11] and the EEGLAB clean_rawdata() pipelines were applied. The PREP pipeline extracts power line frequency at 50 Hz and eliminates artefacts from noisy channels before assigning a robust average reference. The clean_rawdata() pipeline applies a FIR high pass filter of 0.5 Hz (transition band 0.25 - 0.75 Hz, stop-band attenuation 80 dB, order 1286) and removes noisy and flat channels. Additionally, Artifact Subspace Reconstruction [12] was run to reconstruct data segments with abnormally high power (more than 15 standard deviations from the calibration data). If the signal could not be reconstructed, the data segment was removed. A low pass filter was set to 40 Hz (sinc FIR filter, order 86) [13]. A subtraction method [14] rejected ocular artifacts. The Multiple Artifact Rejection Algorithm (MARA) [15,16] performing an independent component analysis identified and removed muscular artifacts and remaining ocular artifacts, as well as loose electrodes. Cardiac artifacts were removed using ICLabel [17]. All missing and removed channels were finally interpolated applying a spherical method.

Section 4: Sample characteristics

A more comprehensive overview of the demographic, questionnaire, and substance-related data collected for each user and control group is provided in Table S1. Please note that hair concentrations

reflect the cumulative value of the specific substance and its metabolites, which were measured by forensic hair toxicology. As METH is metabolised to amphetamine, among others, METH users can achieve high concentrations of amphetamine concentration in hair, although they have not deliberately used amphetamine.

Table S1. Demographic, questionnaire, and substance-related variables for each group per subsample.

	**MDMA control (*n* = 42)**	**MDMA user (*n* = 42)**	**METH control (*n* = 41)**	**METH user (*n* = 38)**	**Group differences**
Sex (female/male)	(24/18)	(22/20)	(12/29)	(12/26)	
Age in years	29.6 ± 1.0	29.9 ± 1.0	30.2 ± 1.0	29.5 ± 1.0	
School education in years	10.1 ± 0.2	10.4 ± 0.2	10.4 ± 0.1	9.6 ± 0.1	MDMA user > METH user * ($t(69.875) = 2.712$; $p_{corr} = .034$)<br>METH control > METH user ** ($t(77) = 3.459$; $p_{corr} = .004$)
Verbal IQ	105.6 ± 1.6	102.1 ± 1.6	104.6 ± 1.3	99.0 ± 1.6	METH control > METH user * ($t(77) = 2.664$; $p_{corr} = .038$)
ADHD total score	10.6 ± 1.1	13.3 ± 1.5	10.0 ± 1.1	15.3 ± 1.6	METH control < METH user * ($t(65.599) = 2.629$; $p_{corr} = .043$)
BIS total score	62.9 ± 1.3	65.2 ± 1.5	60.5 ± 1.0	69.1 ± 1.5	METH control < METH user *** ($t(76) = 4.727$; $p_{corr} < .001$)
CESD total score	9.9 ± 1.2	12.0 ± 1.6	7.1 ± 0.8	18.8 ± 2.2	METH control < METH user *** ($t(48.700) = 4.914$; $p_{corr} < .001$)
STAXI state anger total score	16.0 ± 0.3	17.0 ± 0.9	15.1 ± 0.0	15.6 ± 0.4	
STAXI trait anger total score	17.6 ± 0.5	18.5 ± 0.9	17.8 ± 0.6	19.4 ± 0.9	
Meth craving total score	-	-	-	59.2 ± 1.9	*No statistical comparisons made*
**Alcohol**					
Used in last 12 months	*n = 37*	*n = 42*	*n = 37*	*n = 37*	
Weekly occasions	2.1 ± 0.2	2.2 ± 0.2	1.3 ± 0.2	2.3 ± 0.4	
Weekly amount in gram	94.0 ± 20.2	160.0 ± 27.9	50.1 ± 10.6	143.2 ± 40.9	
Years of use	12.1 ± 1.1	13.8 ± 1.1	13.6 ± 1.0	12.4 ± 1.1	
Cum. lifetime dose in kg	93.5 ± 21.0	158.3 ± 32.1	57.2 ± 11.5	236.9 ± 52.1	METH control < METH user ** ($t(40.627) = 3.364$; $p_{corr} = .007$)
**Nicotine**					
Used in last 12 months	*n = 23*	*n = 30*	*n = 18*	*n = 36*	
Cigarettes per day	2.4 ± 0.7	4.7 ± 0.9	1.7 ± 0.6	12.0 ± 1.0	METH control < METH user *** ($t(63.075) = 8.209$; $p_{corr} < .001$)<br>MDMA user < METH user *** ($t(78) = 5.049$; $p_{corr} < .001$)
Years of use	7.0 ± 1.1	10.1 ± 1.3	6.0 ± 1.0	13.4 ± 1.2	METH control < METH user *** ($t(77) = 4.643$; $p_{corr} < .001$)
**Cannabis**					
Used in last 12 months	*n = 13*	*n = 28*	*n = 11*	*n = 26*	
Weekly occasions	0.2 ± 0.1	0.6 ± 0.2	0.0 ± 0.0	2.1 ± 0.4	METH control < METH user *** ($t(37.216) = 4.671$; $p_{corr} < .001$)<br>MDMA user < METH user * ($t(52.609) = 2.993$; $p_{corr} = .017$)
Weekly amount in gram	0.0 ± 0.0	0.2 ± 0.1	0.0 ± 0.0	1.8 ± 0.4	METH control < METH user ** ($t(37.016) = 3.712$; $p_{corr} = .003$)<br>MDMA user < METH user * ($t(41.152) = 3.079$; $p_{corr} = .015$)
Years of use	4.6 ± 1.0	9.3 ± 1.1	0.6 ± 0.3	8.5 ± 1.2	METH control < METH user *** ($t(43.406) = 6.248$; $p_{corr} < .001$)<br>MDMA control < MDMA user * ($t(82) = 3.029$; $p_{corr} = .013$)<br>METH control < MDMA control ** ($t(51.087) = 3.734$; $p_{corr} = .002$)
Cum. lifetime dose in g	217.3 ± 92.5	752.8 ± 192.6	8.3 ± 5.8	2847.0 ± 938.7	METH control < METH user ** ($t(37.003) = 3.024$; $p_{corr} = .018$)

THC hair concentration in pg/mg	0.0 ± 0.0	0.0 ± 0.0	0.0 ± 0.0	0.0 ± 0.0	MDMA control < MDMA user * (U = 1073.000; p_{corr} = .020)<br>METH control < METH user ** (U = 595.000; p_{corr} = .002)
**MDMA**					
Used in last 12 months	*n = 0*	*n = 42*	*n = 0*	*n = 9*	
Weekly occasions	0.0 ± 0.0	0.3 ± 0.0	0.0 ± 0.0	0.0 ± 0.0	MDMA control < MDMA user *** (t(41.000) = 6.156; p_{corr} < .001)<br>METH user < MDMA user *** (t(41.557) = 5.923; p_{corr} < .001)
Weekly amount in gram	0.0 ± 0.0	0.1 ± 0.0	0.0 ± 0.0	0.0 ± 0.0	MDMA control < MDMA user *** (t(41.000) = 6.234; p_{corr} < .001)<br>METH user < MDMA user *** (t(41.807) = 6.027; p_{corr} < .001)
Years of use	0.0 ± 0.0	8.9 ± 0.8	0.0 ± 0.0	3.2 ± 0.8	MDMA control < MDMA user *** (t(41.000) = 10.268; p_{corr} < .001)<br>METH user < MDMA user *** (t(78) = 4.549; p_{corr} < .001)<br>METH control < METH user ** (t(37.000) = 3.679; p_{corr} = .003)
Cum. lifetime dose in g	0.0 ± 0.0	76.0 ± 17.1	0.0 ± 0.0	33.1 ± 9.4	MDMA control < MDMA user *** (t(41.000) = 4.436; p_{corr} < .001)<br>METH control < METH user ** (t(37.000) = 3.495; p_{corr} < .005)
Days since last use	2774.5 ± 355.5	21.8 ± 2.7	731.0 ± 0.0	1667 ± 331.2	METH user > MDMA user *** (t(32.004) = 4.966; p_{corr} < .001)
Hair concentration in pg/mg	0.0 ± 0.0	809.0 ± 557.5	0.0 ± 0.0	13.0 ± 13.0	MDMA control < MDMA user *** (U = 1721.000; p_{corr} < .001)<br>METH user < MDMA user *** (U = 71.000; p_{corr} < .001)<br>METH control < METH user *** (U = 770.000; p_{corr} < .001)
**METH**					
Used in last 12 months	*n = 0*	*n = 1*	*n = 0*	*n = 38*	
Weekly occasions	0.0 ± 0.0	0.0 ± 0.0	0.0 ± 0.0	3.1 ± 0.3	METH control < METH user *** (t(37.000) = 7.988; p_{corr} < .001)<br>MDMA user < METH user *** (t(37.000) = 7.986; p_{corr} < .001)
Weekly amount in gram	0.0 ± 0.0	0.0 ± 0.0	0.0 ± 0.0	1.5 ± 0.3	METH control < METH user *** (t(37.000) = 5.232; p_{corr} < .001)<br>MDMA user < METH user *** (t(37.000) = 5.232; p_{corr} < .001)
Years of use	0.0 ± 0.0	0.0 ± 0.0	0.0 ± 0.0	9.1 ± 0.8	METH control < METH user *** (t(37.000) = 10.656; p_{corr} < .001)<br>MDMA user < METH user *** (t(37.000) = 10.656; p_{corr} < .001)
Cum. lifetime dose in g	0.0 ± 0.0	0.0 ± 0.0	0.0 ± 0.0	1438.2 ± 287.5	METH control < METH user *** (t(37.000) = 5.002; p_{corr} < .001)<br>MDMA user < METH user *** (t(37.000) = 5.002; p_{corr} < .001)
Days since last use	-	222.0 ± 0.0	-	69.7 ± 16.0	*[only one data point in the MDMA user group]*
Hair concentration in pg/mg	0.0 ± 0.0	0.0 ± 0.0	0.0 ± 0.0	905.0 ± 881.5	METH control < METH user ** (U = 908.000; p_{corr} = .002)<br>MDMA user < METH user *** (U = 1077.000; p_{corr} < .001)
**Amphetamine**					
Used in last 12 months	*n = 0*	*n = 24*	*n = 1*	*n = 10*	
Weekly occasions	0.0 ± 0.0	0.1 ± 0.0	0.0 ± 0.0	0.2 ± 0.1	MDMA control < MDMA user * (t(41.000) = 3.062; p_{corr} = .015)
Weekly amount in gram	0.0 ± 0.0	0.0 ± 0.0	0.0 ± 0.0	0.3 ± 0.2	MDMA control < MDMA user * (t(41.000) = 2.825; p_{corr} = .029)
Years of use	0.0 ± 0.0	4.4 ± 1.0	0.0 ± 0.0	3.4 ± 0.8	METH control < METH user ** (t(37.000) = 3.897; p_{corr} = .001)<br>MDMA control < MDMA user *** (t(41.000) = 4.290; p_{corr} < .001)

Cum. lifetime dose in g	0.0 ± 0.0	63.1 ± 31.6	0.0 ± 0.0	233.8 ± 128.6	
Hair concentration in pg/mg	0.0 ± 0.0	14.0 ± 14.0	0.0 ± 0.0	111.5 ± 106.5	METH control < METH user *** (U = 985.500; p_{corr} < .001)<br>MDMA user < METH user *** (U = 848.000; p_{corr} < .001)<br>MDMA user > MDMA control *** (U = 1312.000; p_{corr} < .001)
**Cocaine**					
Used in last 12 months	*n = 3*	*n = 30*	*n = 1*	*n = 9*	
Weekly occasions	0.0 ± 0.0	0.1 ± 0.0	0.0 ± 0.0	0.0 ± 0.0	MDMA control < MDMA user * (t(41.057) = 3.141; p_{corr} = .012)<br>METH user < MDMA user * (t(44.557) = 2.647; p_{corr} = .045)
Weekly amount in gram	0.0 ± 0.0	0.0 ± 0.0	0.0 ± 0.0	0.0 ± 0.0	MDMA control < MDMA user * (t(41.001) = 2.924; p_{corr} = .022)<br>METH user < MDMA user * (t(41.949) = 2.660; p_{corr} = .044)
Years of use	0.0 ± 0.0	3.7 ± 0.8	0.0 ± 0.0	0.9 ± 0.3	MDMA control < MDMA user *** (t(41.000) = 4.358; p_{corr} < .001)<br>METH user < MDMA user * (t(57.180) = 2.997; p_{corr} = .016)
Cum. lifetime dose in g	0.0 ± 0.0	27.6 ± 7.7	0.0 ± 0.0	17.7 ± 11.7	MDMA control < MDMA user ** (t(41.000) = 3.546; p_{corr} < .004)
Hair concentration in pg/mg	0.0 ± 0.0	162.0 ± 162.0	0.0 ± 0.0	0.0 ± 0.0	MDMA user > METH user *** (U = 242.500; p_{corr} < .001)<br>MDMA user > MDMA control *** (U = 1451.000; p_{corr} < .001)<br>METH control < METH user *** (U = 577.500; p_{corr} = .004)

Note: Substance use in both weekly occasions and weekly amount in gram refer to the last 12 months. Hair data was collected for *n* = 41 MDMA controls, *n* = 42 MDMA users, *n* = 35 METH controls and *n* = 26 METH users. **Because of a strongly positive skewness, values of hair concentrations are given as median ± median absolute deviation (all other values are given as mean ± standard error of the mean)**. Total scores of ADHD, BIS, and CESD have data missing for one METH control. Total scores of STAXI state and trait anger have data missing for three MDMA users. As none of the control participants in either group reported to have ever consumed METH, there is no control group data for days since last (METH) use. *ADHD* = Attention-Deficit/Hyperactivity Disorder; *BIS* = Barratt Impulsiveness Scale; *CESD* = Center for Epidemiologic Studies Depression Scale; *STAXI* = State-Trait Anger Expression Inventory; ***THC*** = tetrahydrocannabinol; * = p < .05; ** = p < .01; *** = p < .001 (all p values were Bonferroni-corrected depending on the number of group comparisons run for the respective variable).

Section 5: Behavioural data

The task-related behavioural data is illustrated in Figure S1. Potential confounders in the overall sample as well as user-specific confounders are further explored below.

5.1. Task effects

For accuracy, the mixed ANOVA yielded a main effect of congruency ($F_{(1,159)}$ = 66.025; p < .001; η_p^2 = 0.293). Participants responded more accurately in congruent (89.7 % ± 0.4) than in incongruent trials (87.7 % ± 0.5). A main effect of valence ($F_{(1,159)}$ = 16.544; p < .001; η_p^2 = 0.094) revealed higher accuracy in happy (89.4 % ± 0.5) than in angry trials (88.0 % ± 0.5). We furthermore obtained an interaction between congruency and valence ($F_{(1,159)}$ = 18.296; p < .001; η_p^2 = 0.103). For each congruency condition, we ran separate post-hoc paired-samples t-tests. In the incongruent condition, participants responded less accurately in angry (86.7 % ± 0.6) than in happy trials (88.9 % ± 0.5) ($t_{(162)}$ = -5.500; p < .001; d = -0.431). No such difference in emotional valence was obtained in the congruent condition ($t_{(162)}$ = -1.471; p = .286). The emotional Stroop effect (i.e., congruent minus incongruent condition), was larger in angry (|2.8| % ± 0.3) than in happy trials (|1.1| % ± 0.3) ($t_{(162)}$ = 4.306; p < .001; d = 0.337).

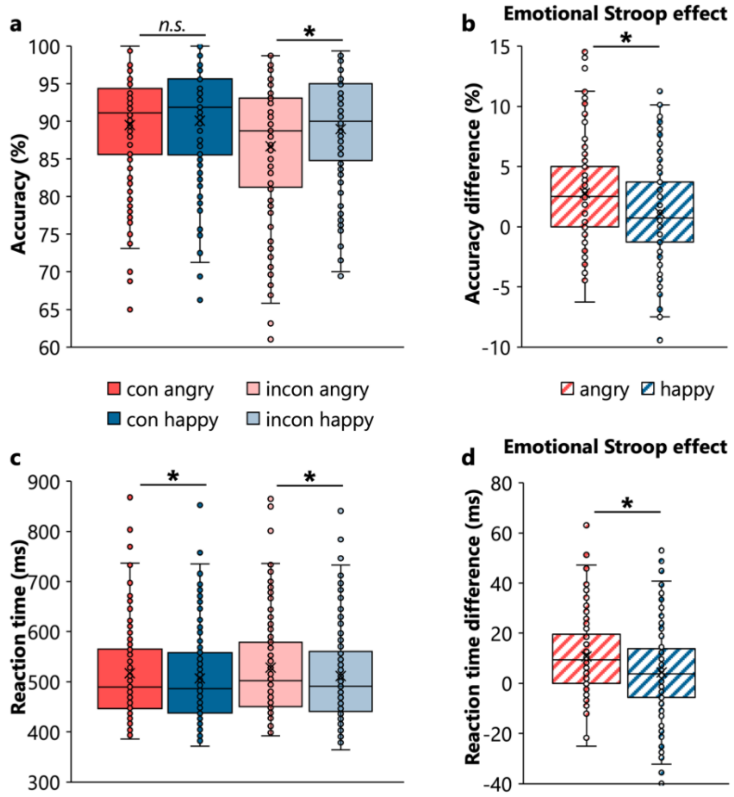

Figure S1. Illustration of the behavioural interaction effects between emotional valence and congruency found for both accuracy (in percent, upper graphs) and reaction time (RT in milliseconds, lower graphs). (**a**) Participants responded significantly less accurately in angry than in happy trials, but only when a cognitive-emotional conflict evolved. (**b**) Thus, the size of the emotional Stroop effect (i.e., congruent minus incongruent trials) was significantly larger in angry than in happy trials. (**c**) Participants responded significantly slower in angry than in happy trials, independently of the congruency condition. (**d**) Nevertheless, the size of the emotional Stroop effect (i.e., incongruent minus congruent) was significantly larger in angry than in happy trials.

For reaction times (RT), the mixed ANOVA showed a main effect of congruency ($F_{(1,159)}$ = 54.294; p < .001; η_p^2 = 0.255). Participants responded faster in congruent (512 ms ± 6.8) than in incongruent trials (519 ms ± 7.3). A main effect of valence ($F_{(1,159)}$ = 57.698; p < .001; η_p^2 = 0.266) showed faster RTs in

happy (509 ms ± 6.9) than in angry trials (522 ms ± 7.3). There was furthermore an interaction between congruency and valence ($F_{(1,159)}$ = 18.371; p < .001; η_p^2 = 0.104). For each congruency condition, we performed separate post-hoc paired-samples t-tests. In the incongruent condition, participants revealed slower responses in angry (528 ms ± 7.7) than in happy trials (511 ms ± 7.2) ($t_{(162)}$ = 8.292; p < .001; d = 0.649). In the congruent condition, participants also responded slower in angry (517 ms ± 7.1) than in happy trials (506 ms ± 6.9) ($t_{(162)}$ = 5.748; p < .001; d = 0.450). However, the emotional Stroop effect (i.e., incongruent minus congruent trials) was larger in angry (10 ms ± 1.1) than in happy trials (4 ms ± 1.3) ($t_{(162)}$ = 4.304; p < .001; d = 0.337).

5.2. Potential confounders in the overall sample on the observed behavioural drug effects

As evident in Table S1, the subsamples differed in school education, ADHD total score, BIS total score, CESD total score, as well as in cumulative lifetime dose of alcohol, years of nicotine use, daily amount of cigarettes, weekly cannabis occasions and amount in the last 12 months, years of cannabis use, and cumulative lifetime use of cannabis. To determine if there were any relationships between these variables and the behavioural measures in our sample, we first conducted exploratory correlational analyses. If there was a significant correlation, we controlled for this potential confounder by re-running the mixed ANOVA as a mixed ANCOVA using the respective factor as co-variate.

For accuracy, the correlational analyses included the accuracy for each condition combination and each valence condition (as we had found a significant interaction effect between valence and group). We observed negative correlations between the CESD total score and each angry condition ($r_{\text{cong-angry}}$ = -0.158, $p_{\text{cong-angry}}$ = .044; $r_{\text{incong-angry}}$ = -0.162, $p_{\text{incong-angry}}$ = .040; $r_{\text{mean-angry}}$ = -0.165, $p_{\text{mean-angry}}$ = .036). Thus, the higher the depression scores, the less accurate participants responded to angry faces (regardless of congruency). We thus included CESD total score as a covariate in the reported ANOVA for accuracy. When controlling for the CESD total score, the interaction effect between group and valence was no longer significant ($F_{(1,157)}$ = 2.499; p = .116). Furthermore, cumulative lifetime dose of alcohol correlated negatively with accuracy in congruent-happy trials (r = -0.158, p = .044). Controlling for lifetime dose of alcohol by running an ANCOVA did however not change the significance of the observed drug effects. Further negative correlations were obtained between cigarettes per day and congruent-angry trials (r = -0.173, p = .027) and mean happy trials (r = -0.155, p = .048), respectively. Yet, all the reported drug effects for accuracy remained significant when controlling for cigarettes per day. Further significant negative correlations were observed between weekly cannabis occasions (in the last 12 months) and all included accuracy measures (-0.271 ≤ r ≤ -0.252, all p ≤ .001). The more often participants used cannabis per week, the less accurate their performance was on the task. When including weekly cannabis occasions as a covariate in the ANOVA, only the main effect of subsample was no longer significant ($F_{(1,157)}$ = 2.888; p = .091). Furthermore, negative correlations were also shown

between weekly cannabis amount (in the last 12 months) and each angry condition ($r_{\text{cong-angry}}$ = -0.158, $p_{\text{cong-angry}}$ = .043; $r_{\text{incong-angry}}$ = -0.166, $p_{\text{incong-angry}}$ = .034; $r_{\text{mean-angry}}$ = -0.168, $p_{\text{mean-angry}}$ = .032). The larger the cannabis amounts used per week, the less accurate participants responded to angry faces (regardless of congruency). When controlling for weekly cannabis amount, only the main effect of subsample was no longer significant, but still at trend level ($F_{(1,157)}$ = 3.837; p = .052). All other correlations with accuracy measures were not significant ($r \leq |0.152|$; $p \geq .053$).

For RT, the correlation analyses included RT variables in each condition combination and in each congruency condition (because of the significant interaction effect between congruency and group). We found a negative correlation between school education and congruent-happy trials (r = -0.160, p = .041). Controlling for school education by including it as a covariate in the RT ANOVA did not change the significance of the observed drug effects. No other correlation reached significance ($r \leq |0.146|$; $p \geq .063$).

5.3. Potential confounders in MDMA and METH users on the observed behavioural and neurophysiological drug effects

Our user groups were to some extent heterogeneous. While we consider this a strength of the study because it is a more realistic reflection of the user populations, we nevertheless examined potential confounding variables within our user groups. For MDMA users, we ran exploratory correlational analyses between relevant behavioural measures and potential confounders, which are detailed in Table S2.

Table S2. Details on included variables for exploratory correlational analyses in MDMA users

**Behavioural measures**	**Potential confounders in MDMA users**
**Accuracy variables (in %)**	1. polystimulant user according to hair analysis (y/n)
congruent-happy	**amount of hair concentration (in pg/mg)**
congruent-angry	2. MDMA
incongruent-happy	3. cocaine
incongruent-angry	4. amphetamine
mean happy	5. cannabis
mean angry	**weekly amount (in g)**
**RT variables (in ms)**	6. MDMA
congruent-happy	7. cocaine
congruent-angry	8. amphetamine
incongruent-happy	9. cannabis
incongruent-angry	**cumulative lifetime dose (in g)**
mean congruent	10. MDMA
mean incongruent	11. cocaine
	12. amphetamine
	13. cannabis
	14. MDMA abstinence duration

Note. *%* = percent; *ms* = milliseconds; *y/n* = yes/no; *pg/mg* = picogram/milligram; *g* = gram.

Importantly, hair concentration of MDMA correlated positively with all RT measures (0.339 ≤ *r* ≤ 0.437; .004 ≤ *p* ≤ .028). This shows that MDMA users performed the task more slowly, as levels of MDMA concentration in hair increased. This was further supported by positive correlations between the weekly amount of MDMA use and RT in congruent happy (*r* = 0.343, *p* = .026), incongruent happy (*r* = 0.324, *p* = .036), and mean congruent trials (*r* = 0.310, *p* = .046). The more MDMA was used per week over the past 12 months, the slower users responded in happy and congruent trials. All other correlations were not significant (*r* ≤ |0.299|; *p* ≥ .055). Given this correlation, we also ran add-on exploratory correlational analyses between the significant MDMA-related variables (i.e., hair concentration of MDMA as well as self-reported weekly intake of MDMA) and the assessed ERPs. This yielded no significant correlations for the P1 (*r* ≤ |0.247|; *p* ≥ .115), the N1 (*r* ≤ |0.189|; *p* ≥ .230), or the N2 (*r* ≤ |0.230|; *p* ≥ .143). For the CSP at electrode CPz, there were significant negative correlations between hair concentration of MDMA and CSP amplitude for congruent angry (*r* = -0.403, *p* = .008), incongruent angry (*r* = -0.439, *p* = .004), and incongruent happy (*r* = -0.311, *p* = .045), but not congruent happy (*r* = -0.292, *p* = .060) trials. All other correlations with the weekly amount of MDMA use were not significant (*r* ≤ |0.227|; *p* ≥ .081). For the P3, there were significant negative correlations between hair concentration of MDMA and P3 amplitude at electrode P3 for incongruent angry trials (*r* = -0.307, *p* = .048). All other correlations with hair concentration of MDMA or the weekly amount of MDMA use were not significant (*r* ≤ |0.282|; *p* ≥ .070).

For METH users, we also ran exploratory correlational analyses between relevant behavioural measures and potential confounders, which are detailed in Table S3. None of the correlations reached significance (*r* ≤ |0.376|; *p* ≥ .059).

Table S3. Details on included variables for exploratory correlational analyses in METH users

**Behavioural measures**	**Potential confounders in METH users**
**Accuracy variables (in %)**<br>congruent-happy<br>congruent-angry<br>incongruent-happy<br>incongruent-angry<br>mean happy<br>mean angry<br>**RT variables (in ms)**<br>congruent-happy<br>congruent-angry<br>incongruent-happy<br>incongruent-angry<br>mean congruent<br>mean incongruent	1. Positive urine result (y/n)<br>2. comorbid psychiatric disorder (y/n)<br>**amount of hair concentration (in pg/mg)**<br>3. METH<br>4. cannabis<br>**weekly amount (in g)**<br>5. METH<br>6. cannabis<br>**cumulative lifetime dose (in g)**<br>7. METH<br>8. cannabis<br>9. METH abstinence duration<br>10. METH craving score at study appointment

Note. % = percent; *ms* = milliseconds; *y/n* = yes/no; *pg/mg* = picogram/milligram; *g* = gram.

Section 6: Task effects reflected in neurophysiological data

The P1, N1, N2, P3, and CSP for each condition combination are illustrated in Figure S2.

For the P1 amplitudes at electrodes P7/P8, the mixed ANOVA revealed a significant interaction between valence and congruency ($F_{(1,159)}$ = 5.869; p = .017; η_p^2 = 0.036). Similarly to the behavioural analysis, we ran separate post-hoc paired-samples t-tests for each congruency condition. In both congruency conditions, the differences in P1 amplitude between angry and happy trials were not significant (all $t \leq |1.646|$; $p \geq .102$). Yet, the P1 Stroop effect (incongruent minus congruent) was larger in happy (|0.6| µV/m^2 ± 0.2) than in angry trials (|0.2| µV/m^2 ± 0.2) ($t_{(162)}$ = 2.269; p = .025; d = 0.178). No other main or interaction effects corresponding to the behavioural task effects emerged (all $F \leq$ 0.707; $p \geq .402$).

For the N1 amplitudes at electrodes P7/P8, we found a main effect of valence ($F_{(1,159)}$ = 83.083; p < .001; η_p^2 = 0.343), showing a larger N1 amplitudes in angry (-17.6 µV/m^2 ± 1.6) than in happy trials (-14.8 µV/m^2 ± 1.5). No other main or interaction effects corresponding to the behavioural task effects were obtained (all $F \leq 2.144$; $p \geq .145$)

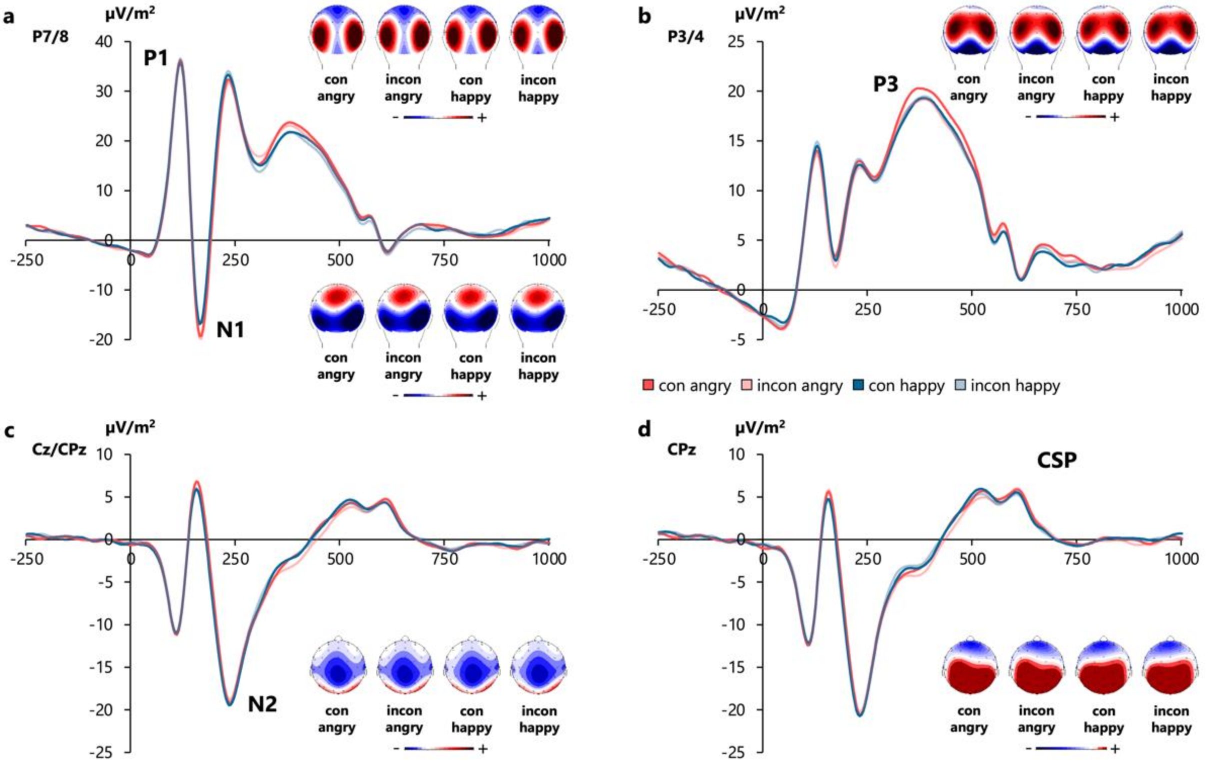

Figure S2. Illustration of ERP components for each condition combination. (**a**) P1 (100 – 130 ms) and N1 (155 – 180 ms), averaged over electrodes P7 and P8. (**b**) P3 (355 – 410 ms), averaged over electrodes P3 and P4. (**c**) N2 (205 – 300 ms) averaged over electrodes Cz and CPz. (**d**) Conflict slow potential (CSP, 490 – 630 ms) at electrode CPz. In all graphs, time point zero marks the stimulus onset, and maps of scalp topography depict the electrical potential in the respective time windows of each ERP component. In the topographies, positivity is marked in red, while negativity is marked in blue.

For the N2 amplitudes averaged at electrodes Cz and CPz, we obtained a main effect of valence ($F_{(1,159)}$ = 6.799; p = .010; η_p^2 = 0.041), showing a higher N2 amplitude in happy (-15.0 µV/m^2 ± 0.6) than in angry trials (-14.5 µV/m^2 ± 0.6). No other main or interaction effects corresponding to the behavioural task effects were observed (all $F \leq 0.452$; $p \geq .503$).

For the CSP amplitudes at electrode CPz, the mixed ANOVA revealed a significant main effect of congruency ($F_{(1,159)}$ = 5.288; p = .023; η_p^2 = 0.032), demonstrating a more pronounced CSP in congruent (5.1 µV/m^2 ± 0.3) than in incongruent trials (4.8 µV/m^2 ± 0.3). No other main or interaction effects corresponding to the behavioural task effects were obtained (all $F \leq 0.174$; $p \geq .677$).

For the P3 amplitudes at electrodes P3/P4, we found a main effect of congruency ($F_{(1,159)}$ = 15.633; p < .001; η_p^2 = 0.090), showing a greater P3 amplitude in congruent (19.5 µV/m^2 ± 0.7) than in incongruent trials (19.0 µV/m^2 ± 0.7). An interaction between congruency and valence was obtained ($F_{(1,159)}$ = 29.757; p < .001; η_p^2 = 0.158). Separate post-hoc paired-samples t-tests for each congruency condition revealed that the P3 amplitude was larger in congruent angry (20.1 µV/m^2 ± 0.8) than in congruent happy trials (19.0 µV/m^2 ± 0.7) ($t_{(162)}$ = 4.460; p < .001; d = 0.349), whereas incongruent angry and incongruent happy trials did not differ significantly ($t_{(162)}$ = -1.128; p = .522). Also, the P3 congruency effect (incongruent minus congruent) was larger in angry (|1.1| µV/m^2 ± 0.1) than in happy trials (|0.1| µV/m^2 ± 0.1) ($t_{(162)}$ = -5.396; p < .001; d = -0.423), which is consistent with the observed emotional Stroop effect in the behavioural data. No other main or interaction effects corresponding to the behavioural task effects emerged (all $F \leq 3.842$; $p \geq .052$).

References

1. Goodwin, R. S. *et al.* Urinary Elimination of 11-Nor-9-carboxy- 9-tetrahydrocannnabinol in Cannabis Users During Continuously Monitored Abstinence. *J Anal Toxicol* **32**, 562–569 (2008).
2. Gouzoulis-Mayfrank, E. & Daumann, J. The confounding problem of polydrug use in recreational ecstasy/MDMA users: a brief overview. *J Psychopharmacol* **20**, 188–193 (2006).
3. Gouzoulis-Mayfrank, E. *et al.* Methamphetamine-Related Disorders. *Dtsch Arztebl Int* **114**, 455–461 (2017).
4. Harro, J. Neuropsychiatric Adverse Effects of Amphetamine and Methamphetamine. *Int Rev Neurobiol* **120**, 179–204 (2015).
5. Etkin, A., Egner, T., Peraza, D. M., Kandel, E. R. & Hirsch, J. Resolving Emotional Conflict: A Role for the Rostral Anterior Cingulate Cortex in Modulating Activity in the Amygdala. *Neuron* **51**, 871–882 (2006).
6. Schreiter, M. L., Chmielewski, W. & Beste, C. Neurophysiological processes and functional neuroanatomical structures underlying proactive effects of emotional conflicts. *Neuroimage* **174**, 11–21 (2018).
7. Schreiter, M. L., Chmielewski, W. X. & Beste, C. How socioemotional setting modulates late-stage conflict resolution processes in the lateral prefrontal cortex. *Cognitive, Affective, & Behavioral Neuroscience* **18**, 521–535 (2018).
8. Tottenham, N. *et al.* The NimStim set of facial expressions: Judgments from untrained research participants. *Psychiatry Res* **168**, 242–249 (2009).
9. Pedroni, A., Bahreini, A. & Langer, N. Automagic: Standardized Preprocessing of Big EEG Data. 460469 Preprint at https://doi.org/10.1101/460469 (2019).
10. Delorme, A. & Makeig, S. EEGLAB: an open source toolbox for analysis of single-trial EEG dynamics including independent component analysis. *Journal of Neuroscience Methods* **134**, 9–21 (2004).
11. Bigdely-Shamlo, N., Mullen, T., Kothe, C., Su, K.-M. & Robbins, K. A. The PREP pipeline: standardized preprocessing for large-scale EEG analysis. *Frontiers in Neuroinformatics* **9**, (2015).
12. Mullen, T. *et al.* Real-time modeling and 3D visualization of source dynamics and connectivity using wearable EEG. in *2013 35th Annual International Conference of the IEEE Engineering in Medicine and Biology Society (EMBC)* 2184–2187 (2013). doi:10.1109/EMBC.2013.6609968.
13. Widmann, A., Schröger, E. & Maess, B. Digital filter design for electrophysiological data – a practical approach. *Journal of Neuroscience Methods* **250**, 34–46 (2015).
14. Parra, L. C., Spence, C. D., Gerson, A. D. & Sajda, P. Recipes for the linear analysis of EEG. *NeuroImage* **28**, 326–341 (2005).
15. Winkler, I., Haufe, S. & Tangermann, M. Automatic Classification of Artifactual ICA-Components for Artifact Removal in EEG Signals. *Behavioral and Brain Functions* **7**, 30 (2011).
16. Winkler, I. *et al.* Robust artifactual independent component classification for BCI practitioners. *J. Neural Eng.* **11**, 035013 (2014).
17. Pion-Tonachini, L., Kreutz-Delgado, K. & Makeig, S. The ICLabel dataset of electroencephalographic (EEG) independent component (IC) features. *Data in Brief* **25**, 104101 (2019).
